# Evaluation of State Cannabis Laws and Rates of Self-harm and Assault

**DOI:** 10.1001/jamanetworkopen.2021.1955

**Published:** 2021-03-18

**Authors:** Ellicott C. Matthay, Mathew V. Kiang, Holly Elser, Laura Schmidt, Keith Humphreys

**Affiliations:** 1 Center for Health and Community, University of California, San Francisco; 2 Department of Epidemiology and Population Health, Stanford University School of Medicine, Palo Alto, California; 3 Medical student, Stanford University School of Medicine, Palo Alto, California; 4 Philip R. Lee Institute for Health Policy Studies and Department of Humanities and Social Sciences, University of California, San Francisco; 5 Center for Innovation to Implementation, Veterans Affairs Palo Alto Health Care System, Palo Alto, California; 6 Department of Psychiatry and Behavioral Sciences, Stanford University School of Medicine, Palo Alto, California

## Abstract

**Importance:**

State cannabis laws are changing rapidly. Research is inconclusive about their association with rates of self-harm and assault. Existing studies have not considered variations in cannabis commercialization across states over time.

**Objective:**

To evaluate the association of state medical and recreational cannabis laws with self-harm and assault, overall and by age and sex, while considering varying degrees of commercialization.

**Design, Setting, and Participants:**

Using a cohort design with panel fixed-effects analysis, within-state changes in claims for self-harm and assault injuries before and after changes in cannabis laws were quantified in all 50 US states and the District of Columbia. Comprehensive claims data on commercial and Medicare Advantage health plan beneficiaries from January 1, 2003, to December 31, 2017, grouped by state and month, were evaluated. Data analysis was conducted from January 31, 2020, to January 21, 2021.

**Exposures:**

Categorical variable that indexed the degree of cannabis legalization in each state and month based on law type (medical or recreational) and operational status of dispensaries (commercialization).

**Main Outcomes and Measures:**

Claims for self-harm and assault injuries based on *International Classification of Diseases* codes.

**Results:**

The analysis included 75 395 344 beneficiaries (mean [SD] age, 47 [22] years; 50% female; and median follow-up, 17 months [interquartile range, 8-36 months]). During the study period, 29 states permitted use of medical cannabis and 11 permitted recreational cannabis. Point estimates for populationwide rates of self-harm and assault injuries were higher in states legalizing recreational cannabis compared with states with no cannabis laws, but these results were not statistically significant (adjusted rate ratio [aRR] assault, recreational dispensaries: 1.27; 95% CI, 0.79-2.03;self-harm, recreational dispensaries aRR: 1.15; 95% CI, 0.89-1.50). Results varied by age and sex with no associations found except for states with recreational policies and self-harm among males younger than 40 years (aRR <21 years, recreational without dispensaries: 1.70; 95% CI, 1.11-2.61; aRR aged 21-39 years, recreational dispensaries: 1.46; 95% CI, 1.01-2.12). Medical cannabis was generally not associated with self-harm or assault injuries populationwide or among age and sex subgroups.

**Conclusions and Relevance:**

Recreational cannabis legalization appears to be associated with relative increases in rates of claims for self-harm among male health plan beneficiaries younger than 40 years. There was no association between cannabis legalization and self-harm or assault, for any other age and sex group or for medical cannabis. States that legalize but still constrain commercialization may be better positioned to protect younger male populations from unintended harms.

## Key Points

**Question:**

Are state cannabis legalization laws with varying degrees of commercialization associated with rates of self-harm or assault injuries?

**Findings:**

In this cohort study based on health insurance claims data from 75 395 344 beneficiaries between 2003 and 2017, rates of self-harm injuries among males younger than 40 years increased more in states legalizing recreational cannabis dispensaries compared with states without cannabis legalization laws. For other age and sex groups and for more restrictive legalization approaches, no association with self-harm and assault was found.

**Meaning:**

States with recreational cannabis may benefit from monitoring levels of self-harm as a potential consequence of legalization.

## Teaser

This cohort study examines the incidence of self-harm and assault injuries in the US since the legalization of medical as well as recreational use of cannabis.

## Introduction

Cannabis laws may be underappreciated factors for the rates of self-harm and assault,^1,2,3^ the second and fifth leading causes of death among individuals aged 15 to 49 years in the US, respectively.^4^ Since 1996, 36 states and the District of Columbia have legalized cannabis for medical use, and 15 states and the District of Columbia have legalized cannabis for recreational use. Increases in the availability of cannabis products are likely to increase cannabis use, including heavy use.^5,6,7,8,9^ Cannabis intoxication can lead to behavioral disinhibition, altered perceptions and judgment, impaired memory, and elevated physiologic arousal, including paranoia. Collectively, these factors can heighten violence proclivity and increase vulnerability to violence.^10,11,12,13,14,15^ Regular and heavy use of increasingly high-potency cannabis products^16,17^ have also been linked to risk factors for self-harm and assault injury, including impaired cognitive function, concomitant alcohol use, psychosis, depressive disorders, and suicidal ideation and attempts.^10,18^ Meta-analyses of data on youth, intimate partners, and individuals with serious mental disorders, as well as animal and brain imaging studies, link high tetrahydrocannabinol (THC) doses to aggression and violence perpetration.^15^ Furthermore, cannabis dispensaries, like liquor stores, could attract crime in immediately surrounding areas,^18,19,20^ although some have proposed that cannabis legalization could reduce violence by replacing illegal markets and reducing alcohol consumption.^21^

Studies of the population-level association between cannabis legalization and violence-related outcomes (violent crime and suicide) have focused almost exclusively on medical cannabis and yielded conflicting results.^19,20,22,23,24,25,26,27,28,29,30,31,32,33,34^ To our knowledge, no studies have examined assault injuries or nonfatal self-harm, and few have considered associations within age or sex subgroups that may be affected in different ways by cannabis laws. One reason that the findings of previous studies may conflict is that many studies use crude measures of cannabis laws.^35,36^ Yet state cannabis laws vary substantially. Some states permit only small amounts of homegrown cannabis plants for medical use. Others have extensive commercialization, permitting multi-billion dollar, competitive retail markets based on systems of dispensaries offering diverse, high-potency products to anyone aged 21 years or older.^37^ Provisions affecting consumer THC doses may be especially relevant for violence-related outcomes.^15^ These policy differences matter: medical and recreational cannabis legalization per se are not consistently associated with population-level cannabis outcomes, but rates of cannabis use and poison control center calls for cannabis exposures have increased when states permit retail sales through dispensaries.^35,38^

Therefore, in this retrospective cohort study leveraging a panel fixed-effects design,^39^ we evaluated whether varying degrees of state medical and recreational cannabis commercialization were associated with rates of self-harm and assault injuries. In addition, because cannabis use and risk of violent injury vary by age and sex, we tested whether these factors modify associations between cannabis commercialization and violent injury.^23,40^

## Methods

### Setting, Participants, and Procedures

In this cohort study, we obtained data on nonfatal self-harm and assault injuries from a deidentified database (Clinformatics Data Mart; Optum Inc) covering all 50 US states and the District of Columbia from January 1, 2003, to December 31, 2017 (before and after changes in cannabis laws were quantified in all 50 US states and the District of Columbia). The data set included commercial and Medicare Advantage claims for 75 395 344 individuals.^41^ These data have been used extensively to assess health outcomes.^42,43,44,45^ Data included member enrollment dates and inpatient and outpatient claims with service dates and diagnostic codes deterministically linked using a unique patient identifier.^41^ We included individuals of all ages eligible for the insurance product for at least 1 month and residing in any US state or the District of Columbia (hereafter, *states*).

We merged the claims records by state and month with data on recreational cannabis laws from the Alcohol Policy Information System cannabis law database^46,47^ and medical laws from published research.^48,49^ Complete law data were available through 2017. We also linked time-varying state-level covariates on demographic factors, social and economic conditions, and other relevant laws from public sources, including the Census Bureau, Bureau of Labor Statistics, and literature (eTable 1 in the Supplement). Data analysis was conducted from January 31, 2020, to January 21, 2021.

This study followed the Strengthening the Reporting of Observational Studies in Epidemiology (STROBE) reporting guidelines for cohort studies and was approved by the Stanford University Institutional Review Board with a waiver of informed consent because the claims records were deidentified, the research could not practically be carried out without the requested waiver, and the research involved no more than minimal risk to participants.

### Measures

Self-harm and assault injuries were identified as 1 or more health insurance claims during a single day for the treatment of injuries, with an *International Classification of Diseases, Ninth Revision* and *International Statistical Classification of Diseases and Related Health Problems, Tenth Revision* code indicating self-harm or assault (eTable 2 in the Supplement). Self-harm included intentional nonsuicidal self-harm (eg, cutting) and suicide attempts (eg, intentional drug overdose). Assault included injuries intentionally inflicted by one person on another (eg, intimate partner violence). We aggregated counts of self-harm and assault injuries to the state-month level and calculated the rates of claims per insured beneficiaries in that state for each month.

The exposure was a categorical variable reflecting the degree of cannabis commercialization in each state-month. We classified each state and month based on the type of law (medical or recreational) and the availability of dispensaries (commercialization) as follows^35,37,38^: (1) no cannabis legalization law (reference category), (2) medical only without dispensaries, (3) medical only with dispensaries, (4) recreational without dispensaries, and (5) recreational with dispensaries.

States without dispensaries were those that permitted only homegrown cannabis or planned to permit dispensaries but had not yet implemented retail sales. All states that permitted recreational cannabis also permitted medical cannabis. We did not consider decriminalization of use because the focus of this analysis was on state laws permitting cannabis production and sale.

State-level control variables were percentage of non-Hispanic Asian, non-Hispanic Black, non-Hispanic White, and Hispanic race/ethnicity; people living in poverty, unemployed, and renting their housing; median income; overall claims rate; alcohol law stringency (based on 29 best practices^50^); opioid overdose mortality rate; and an indicator for the October 1, 2015, shift from the 9th to 10th revision of the *International Classification of Diseases*. For self-harm analyses, additional controls were percentage of females aged 15 to 29 years (highest risk group), females aged 30 to 44 years, and veterans of military service. For assault analyses, additional controls were percentage of males aged 15 to 29 years (highest risk group), males aged 30 to 44 years, and the violent crime rate. All covariates except the *International Classification of Diseases* shift were lagged by 1 year to ensure temporal ordering.

### Statistical Analysis

The analytic data set included 9180 state-months. We used negative binomial regression to model rates of self-harm and assault separately as a function of cannabis commercialization, controlling for state, month, and year fixed effects and time-varying state-specific covariates. Statistical testing of the dispersion parameters indicated that negative binomial regression models were more appropriate than Poisson regression models. We used cluster robust SEs to account for repeated observations on states over time. State fixed effects controlled for unmeasured characteristics of states that changed little over the study period (eg, provider network characteristics and religiosity) and have consistent effects on injury rates. Year fixed effects controlled for secular changes and events affecting all places (eg, economic recession and increasing THC potency of cannabis plants). Month fixed effects accounted for seasonality in self-harm and assault. The remaining control variables included in the regression models were time-varying, state-specific factors plausibly associated with cannabis commercialization and risk factors for self-harm or assault. In this fixed-effects approach, each place serves as its own control; unmeasured time-invariant characteristics of states and place-invariant characteristics of time are controlled by design.^39^

Initial analyses evaluated the population overall. Secondary analyses stratified by sex (male, female) and age. We defined 4 age categories based on the typical threshold for legal use of cannabis, the age after which risk of violent injury substantially declines, and the age at which health insurance coverage commonly shifts^51^: younger than 21 years, 21 to 39 years, 40 to 64 years, and 65 years and older.

We tested 2 alternative definitions of the denominator used for outcome rates: unique claims (number of unique claims per state-month) to account for changes in overall use and active members (number of unique members filing at least 1 claim per state-month), an approach that is more robust to members with high health care use. We also tested models controlling for firearm availability, measured as the percentage of suicides completed with a firearm (excluding the District of Columbia owing to missing data),^52,53^ and models adjusting for state-specific linear time trends.

Although no states explicitly regulated potency, we hypothesized that THC dose may affect the association between cannabis commercialization and violent injury.^15,35,37^ Thus, we tested a 6-category version of the exposure variable that separated states with recreational dispensaries into those with and without THC dose-related restrictions (defined as limiting any of the following: THC dose per serving size, THC content per package, or product types, eg, bans on edible products).^46,47^ We also tested a 3-category exposure variable (no law vs any medical law vs any recreational law) and conducted analyses restricted to state-months with some form of cannabis legalization law (medical laws without dispensaries was the reference category).

To confirm that results were not an artifact of residual temporal autocorrelation, we used the overall claims rate as a negative control outcome and a hypothetical law change at randomly assigned dates as a negative control exposure. To test for residual confounding by common determinants of drug policy reforms and violent injury, we used naloxone overdose prevention laws as a negative control exposure. Power calculations are presented in the eMethods of the Supplement. Findings were considered significant for unpaired, 2-sided tests at the .05 level. Statistical analysis was performed with R, version 4.0.2 (R Project for Statistical Computing).

## Results

**Figure 1.**
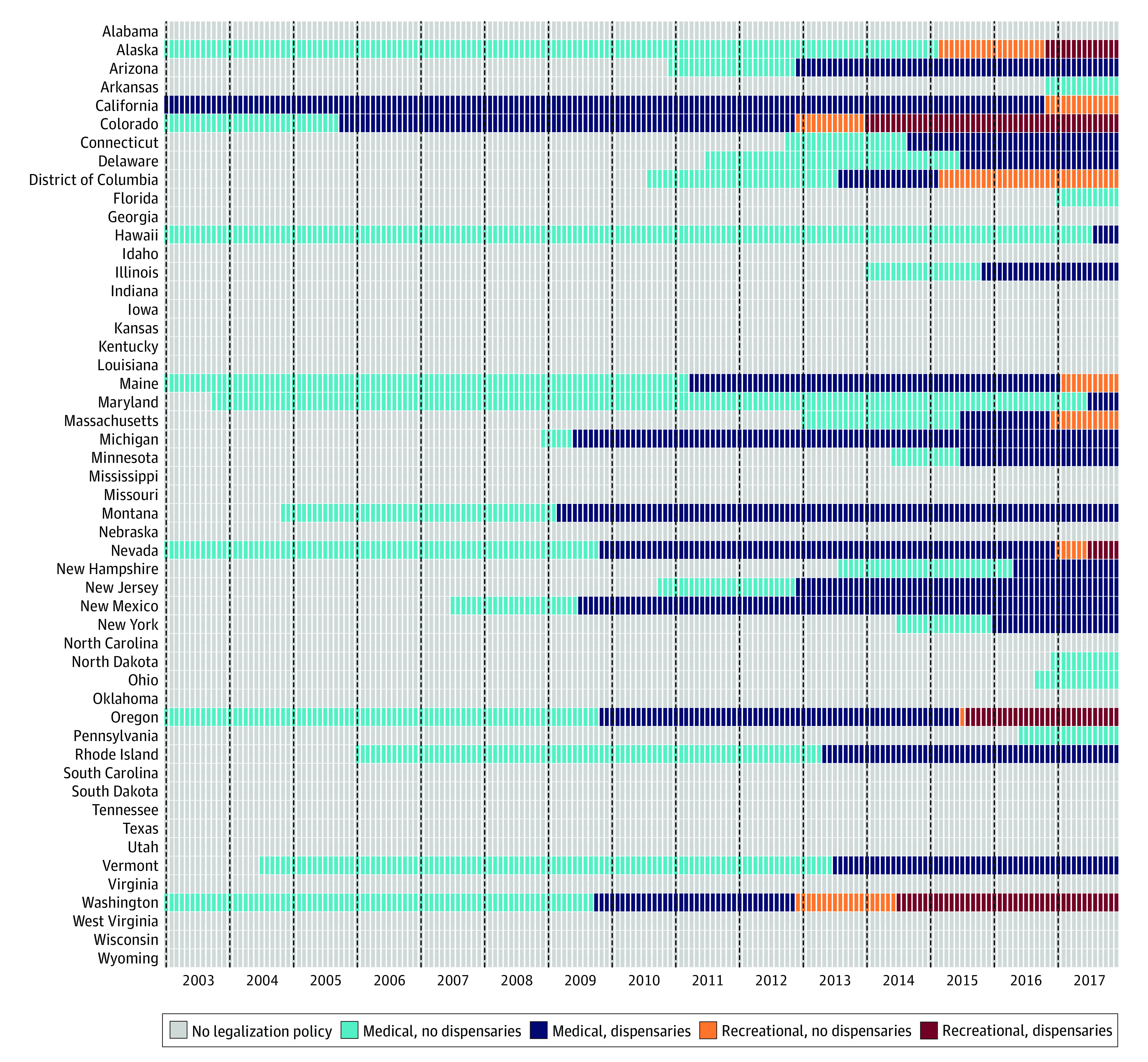
Classification of Medical and Recreational Cannabis Commercialization by State, 2003-2017

The analysis included 75 395 344 beneficiaries (mean [SD] age, 47 [22] years; 50% female, and median follow-up, 17 months [interquartile range, 8-36 months]). The Table presents the characteristics of the study population overall and for each level of cannabis commercialization. eTable 3 in the Supplement presents characteristics of states and months of the study overall and for each level of cannabis commercialization. Figure 1 shows the progression of the exposure, cannabis commercialization from 2003 to 2017. Twenty-nine states had some form of cannabis legalization during the study period and most adopted several revisions. By December 31, 2017, 11 states had adopted recreational laws: 4 without active dispensaries (California, District of Columbia, Maine, and Massachusetts) and 5 with active dispensaries (Alaska, Colorado, Nevada, Oregon, and Washington).

**Table.** Characteristics of Study Population, Overall and by Cannabis Policy Category

Characteristic	National	No legalization policy	Medical, no dispensaries	Medical, dispensaries	Recreational, no dispensaries	Recreational, dispensaries
Beneficiary-months of observation, No. (%)	2 412 798 613 (100)	1 673 543 948 (69)	206 695 016 (9)	458 816 716 (19)	33 676 864 (1)	40 066 069 (2)
State-months of observation, No. (%)	9180 (100)	6144 (67)	1469 (16)	1259 (14)	125 (1)	138 (2)
States contributing time at risk, No. (%)	51 (100)	43 (84)	28 (55)	23 (45)	8 (16)	5 (10)
Self-harm injury claim rate per 100 000 persons, annualized (95% CI)	79.5 (79.0-80.0)	75.4 (74.2-76.6)	73.0 (72.7-73.3)	95.1 (92.5-97.7)	110.8 (109.4-112.2)	162.8 (161.8-163.8)
Assault injury claim rate per 100 000 persons, annualized (95% CI)	93.9 (92.8-95.0)	96.9 (95.5-98.3)	92.5 (92.1-92.9)	80.5 (78.1-82.9)	106.5 (105.1-107.9)	91.8 (91.1-92.5)
Aged 15-29 y, %						
Male sex	9.0	9.4	8.2	8.2	8.9	8.0
Female sex	8.9	9.3	8.3	7.9	8.9	7.6
Overall claims rate per 100 000 persons, annualized (95% CI)	802 417 (801 491-803 343)	772 958 (771 857-774 059)	842 175 (841 831-842 519)	878 621 (876 427-880 815)	897 350 (896 067-898 633)	869 423 (868 729-870 117)

**Figure 2.**
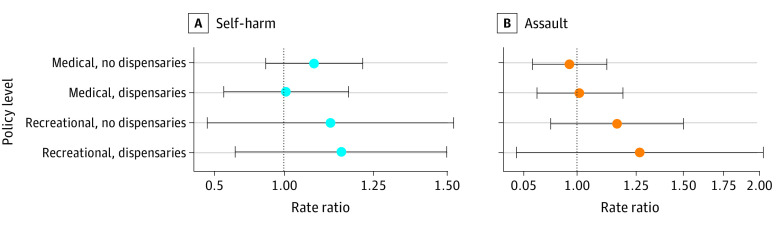
Adjusted Results for Self-harm and Assault Injury Rates by Level of Cannabis Commercialization for the Overall Study Population, 2003-2017 Points represent the adjusted rate ratio for self-harm (A) or assault (B) injuries relative to state-months not adopting any medical or recreational cannabis legalization over the same period. Bars represent the corresponding 95% CIs.

Figure 2 presents the adjusted rate ratios for self-harm and assault injuries across the entire study population. Medical cannabis laws showed no association with self-harm or assault. Point estimates for both outcomes were higher in states with recreational laws, but the findings were not statistically significant (assault, recreational dispensaries: adjusted rate ratio [aRR], 1.27; 95% CI, 0.79-2.03; self-harm, recreational dispensaries: aRR, 1.15; 95% CI, 0.89-1.50).

Overall, the results of sensitivity analyses were consistent with the main results (eFigures 1-11 in the Supplement). The aRRs for recreational dispensaries without THC dose-related restrictions were slightly higher for self-harm and slightly lower for assault compared with those for recreational dispensaries with dose-related restrictions but not statistically significant (eFigure 4 in the Supplement). Examining only medical and recreational policies without considering commercialization resulted in aRRs that were higher than for recreational dispensaries (eFigure 5 in the Supplement). As expected, the results of negative control exposures and outcomes were consistently null (eFigures 6-8 in the Supplement).

**Figure 3.**
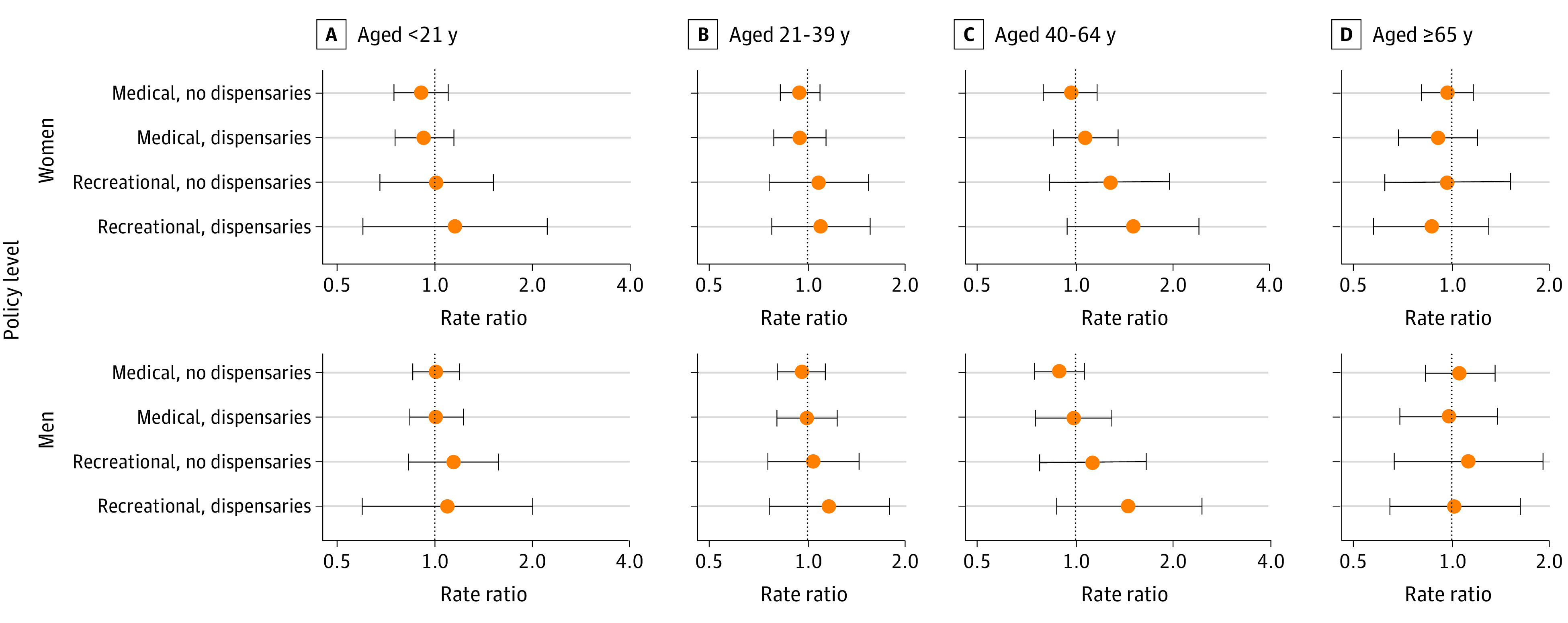
Adjusted Results for Assault Injury Rates at Varying Levels of Cannabis Commercialization, by Age Group and Sex, 2003-2017 Points represent the adjusted rate ratio for assault injuries relative to state-months not adopting any medical or recreational cannabis legalization over the same period. Bars represent the corresponding 95% CIs.

**Figure 4.**
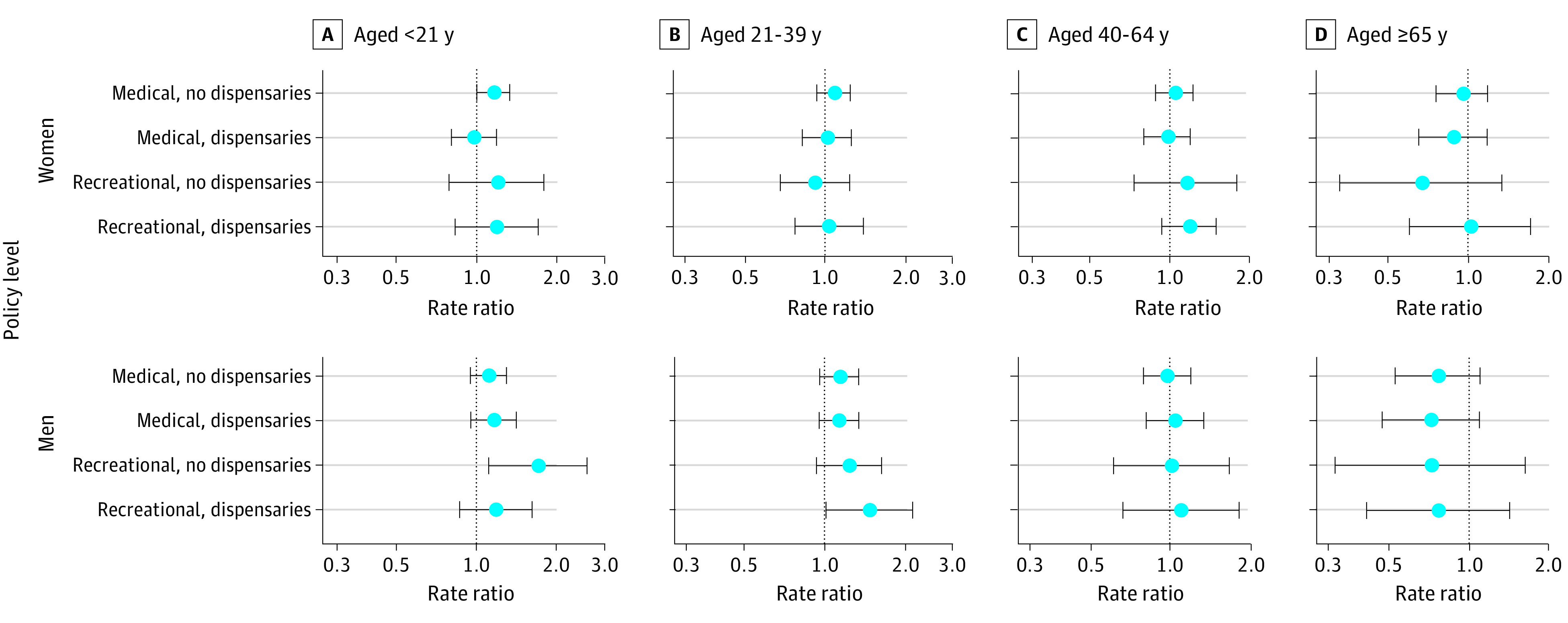
Adjusted Results for Self-harm Injury Rates at Varying Levels of Cannabis Commercialization, by Age Group and Sex, 2003-2017 Points represent the adjusted rate ratio self-harm injuries relative to state-months not adopting any medical or recreational cannabis legalization over the same period. Bars represent the corresponding 95% CIs.

Results by age and sex for assault (Figure 3) and self-harm (Figure 4) are shown. Cannabis laws were generally not associated with assault rates. For adults aged 40 to 64 years, findings were patterned with less restrictive policies indicating larger increases in assault injuries (women aged 40-64 years, recreational dispensaries: aRR, 1.51; 95% CI, 0.94-2.40; men aged 40-64 years, recreational dispensaries: aRR, 1.46; 95% CI, 0.87-2.43). Associations for self-harm varied by age group, sex, and level of cannabis commercialization but were generally negligible. Rates of self-harm injury for young males were associated with recreational cannabis states (males aged <21 years, recreational without dispensaries: aRR, 1.70; 95% CI, 1.11-2.61; males aged 21-39 years, recreational dispensaries: aRR, 1.46; 95% CI, 1.01-2.12). These findings corresponded to an additional 67 self-harm injuries per 100 000 persons per year over baseline (eTable 4 in the Supplement). Men aged 65 years and older had lower rates of self-harm in states with recreational cannabis, but these results were not significant..

The results of sensitivity analyses by age and sex were generally consistent with those of the primary analyses by age and sex (eFigures 12-29 in the Supplement). Recreational cannabis laws permitting dispensaries and lacking dose-related restrictions were associated with significant increases in assault for males and females younger than 21 years (females: aRR, 2.14; 95% CI, 1.33-3.43; males: aRR, 2.29; 95% CI, 1.34-3.92) and increases in self-harm for males aged 21 to 39 years (aRR, 2.27; 95% CI, 1.76-2.93) compared with no medical or recreational cannabis laws.

## Discussion

We found little evidence of an association between state cannabis legalization and populationwide rates of self-harm or assault injuries. However, there were relative increases in rates of self-harm injuries among males younger than 40 years in states with legalized recreational cannabis compared with states without legalized cannabis. To our knowledge, this study is the first to test the association of state cannabis commercialization with nonfatal self-harm and assault injuries. Given the lack of randomization, the findings highlight only state-level associations; both significant and nonsignificant results should be subject to further investigation. Replication studies will be useful if they have detailed individual-level, longitudinal data on substance use and violent injury.

The risks and benefits of cannabis legalization have been debated as if all legalization laws were the same,^54^ but our results suggest that more nuance in characterizing cannabis policies is warranted. Researchers should evaluate not only whether a state has a medical and recreational cannabis law but also whether retail sales are occurring through dispensaries. Although some estimates were imprecise, increases in self-harm and assault associated with cannabis laws were larger for states permitting medical and recreational dispensaries compared with those with legalization laws not permitting dispensaries. This finding is consistent with previous research reporting that increases in cannabis consumption were associated with laws permitting medical dispensaries but not medical laws alone,^35^ and increases in poison control center calls for cannabis exposures were associated with laws permitting recreational dispensaries but not recreational laws alone.^38^ Unlike homegrown cannabis sources, dispensaries imply opportunities for large-scale, profit-driven commercial markets; greater exposure to advertising; and greater access to diverse, rapidly evolving, higher-potency products.^1,37^ In this study, the absence of dose-related product restrictions among states with recreational dispensaries was associated with larger increases in violent injuries. Although no states directly cap the potency of cannabis products, nationwide increases in cannabis potency may disproportionately affect states allowing greater access to cannabis products.^16,17^ Future studies should consider the operational status of dispensaries as well as related provisions related to advertising, product potency and dose, product packaging and labeling, and permitted product types, which vary within states over time.

We observed increases in violent injuries associated with recreational cannabis in populations younger than 21 years, which is the legal age to attain recreational cannabis. Cannabis use is an independent risk factor for physical violence perpetration in young people.^55^ Alcohol research shows that individuals younger than the legal purchasing age are affected by alcohol regulations^56^; similarly, recreational cannabis laws that promote commercialization could make cannabis more accessible for youth despite age limits. Cannabis use in young people may also be a marker of concomitant use with other substances linked to assault and self-harm, including alcohol, amphetamines, cocaine, phencyclidine, and benzodiazepines.^11,57^ Increased cannabis use by adults in legalization states could also contribute to higher rates of youth victimization.^58^

Self-harm injuries associated with recreational cannabis appear to be concentrated in young males. Young males use cannabis more frequently^59^ and increase cannabis use more frequently under legalization than females and older males.^60^ Psychotic disorder, which has been linked to heavy cannabis use, is also a risk factor for self-harm and assault victimization and disproportionately affects men.^18,61^ Future research should evaluate whether increases in self-harm identified in this study can be associated with increases in cannabis use, cannabis use disorder, or associated mental disorders.

### Limitations

Limitations of the study include possible residual confounding by factors that changed in tandem with cannabis commercialization. We minimized the impact of potential confounders by using each state as its own control and adjusting for a range of control variables. We performed negative control analyses to test for unobserved confounders and residual temporal autocorrelation, but alternative explanations for our findings cannot be ruled out. Our study sample is well defined, demographically diverse, and constitutes a large percentage of all privately insured individuals in the US but is not nationally representative and, as an insured population, may underrepresent those at greatest risk for violent injuries. Not all cases of self-harm or assault are documented accurately in health insurance claims, although claims can still serve as a marker. Cause-of-injury coding in most states is subject to ongoing quality assurance procedures,^62^ and misclassification is typically not substantial enough to alter major patterns.^63,64^ Although our study was well powered to detect meaningful associations (eMethods in the Supplement), violent injuries are rare, and some estimates were imprecise. Future years of observation will enhance precision. Coding of state laws is complex; we relied on published sources that rigorously evaluated the legal text, but misclassification cannot be ruled out. Some potential state and individual modifiers were not observed, such as the concomitant use of alcohol and cannabis, which may confer greater risks than the use of either substance alone^65^; cannabis commercialization may be most relevant to self-harm and assault when alcohol concomitant use is likely.^66,67,68^

## Conclusions

This study provides preliminary evidence that violent injuries in young males may be associated with state cannabis laws. Public health officials and clinicians in states with cannabis laws, particularly those permitting recreational cannabis dispensaries, should monitor for increases in violent injuries, particularly self-harm injuries among young males. Future research is needed to verify these findings for populations not captured by insurance claims, for specific kinds of injuries (eg, nonsuicidal self-harm, intimate partner violence, and community violence), and to more precisely document whether and how associations between cannabis commercialization and increases in self-harm or assault injuries are causal. Until such evidence is available, lawmakers should consider provisions consistent with a precautionary principle approach that restrains excessive commercialization, following tobacco and alcohol control policies. Public health best practices include bans on specific products and fraudulent medical cannabis advertising, labeling and packaging requirements, potency and price controls, limits on dispensary densities and locations, public health messaging about safer use, and investments in mental health services.^1,69,70,71,72^ Local variation in cannabis regulatory approaches offers opportunities to efficiently test the outcomes of alternative regulatory approaches on public health.

## Notes

Supplement. **eMethods.** Power Calculations **eTable 1.** Covariate Data Sources and Information **eTable 2.** International Classification of Diseases Clinical Modification (ICD-CM) 9th and 10th Revision Cause of Injury Codes Used to Identify and Classify Self-harm and Assault Injuries **eTable 3.** Characteristics of Study State-Months Overall and by Policy Type **eTable 4.** Beneficiary-Months of Observation and Baseline Self-harm and Assault Injury Rates for Age and Gender Subgroup Analyses **eFigure 1.** Adjusted Associations of Medical and Recreational Cannabis Commercialization With Self-harm and Assault Injury Rates, Using Unique Claims as the Denominator for Outcome Rates, for the Overall Study Population, 2003-2017 **eFigure 2.** Adjusted Associations of Medical and Recreational Cannabis Commercialization With Self-harm and Assault Injury Rates, Using Active Members as the Denominator for Outcome Rates, for the Overall Study Population, 2003-2017 **eFigure 3.** Adjusted Associations of Medical and Recreational Cannabis Commercialization With Self-harm and Assault Injury Rates, Adjusted for Firearm Availability, Without the District of Columbia, for the Overall Study Population, 2003-2017 **eFigure 4.** Adjusted Associations of Medical and Recreational Cannabis Commercialization With Self-harm and Assault Injury Rates, With Recreational Dispensary Categories Disaggregated by THC Dosage-Related Restrictions, for the Overall Study Population, 2003-2017 **eFigure 5.** Adjusted Associations of Medical and Recreational Cannabis Commercialization With Self-harm and Assault Injury Rates, With Medical and Recreational Policy Categories Collapsed, for the Overall Study Population, 2003-2017 **eFigure 6.** Adjusted Associations of Medical and Recreational Cannabis Commercialization With the All-Claims Rate as a Negative Control Outcome, for the Overall Study Population, 2003-2017 **eFigure 7.** Adjusted Associations of Naloxone Overdose Prevention Laws as a Negative Control Exposure With Self-harm and Assault Injury Rates, for the Overall Study Population, 2003-2017 **eFigure 8.** Adjusted Associations of Hypothetical, Randomly Generated Law Changes as a Negative Control Exposure With Self-harm and Assault Injury Rates, for the Overall Study Population, 2003-2017 **eFigure 9.** Adjusted Associations of Medical and Recreational Cannabis Commercialization With Self-harm and Assault Injury Rates, With Additional Adjustment for Linear State-Specific Time Trends, for the Overall Study Population, 2003-2017 **eFigure 10.** Adjusted Associations of Medical and Recreational Cannabis Commercialization With Self-harm and Assault Injury Rates, for States With Cannabis Legalization, for the Overall Study Population, 2003-2017 **eFigure 11.** Adjusted Associations of Recreational (Versus Medical) Cannabis Legalization and Commercialization (Dispensaries Versus Home-Grown Only) With Self-harm and Assault Injury Rates, for States With Cannabis Legalization, for the Overall Study Population, 2003-2017 **eFigure 12.** Adjusted Associations of Medical and Recreational Cannabis Commercialization With Self-harm Injury Rates, Using Unique Claims as the Denominator for Outcome Rates, by Age Group and Gender, 2003-2017 **eFigure 13.** Adjusted Associations of Medical and Recreational Cannabis Commercialization With Self-harm Injury Rates, Using Unique Claims as the Denominator for Outcome Rates, by Age Group and Gender, 2003-2017 **eFigure 14.** Adjusted Associations of Medical and Recreational Cannabis Commercialization With Assault Injury Rates, Using Active Members as the Denominator for Outcome Rates, by Age Group and Gender, 2003-2017 **eFigure 15.** Adjusted Associations of Medical and Recreational Cannabis Commercialization With Self-harm Injury Rates, Using Active Members as the Denominator for Outcome Rates, by Age Group and Gender, 2003-2017 **eFigure 16.** Adjusted Associations of Medical and Recreational Cannabis Commercialization With Assault Injury Rates, Adjusted for Firearm Availability, Without the District of Columbia, by Age Group and Gender, 2003-2017 **eFigure 17.** Adjusted Associations of Medical and Recreational Cannabis Commercialization With Self-harm Injury Rates, Adjusted for Firearm Availability, Without the District of Columbia, by Age Group and Gender, 2003-2017 **eFigure 18.** Adjusted Associations of Medical and Recreational Cannabis Commercialization With Assault Injury Rates, With Recreational Dispensary Categories Disaggregated by THC Dosage-Related Restrictions, by Age Group and Gender, 2003-2017 **eFigure 19.** Adjusted Associations of Medical and Recreational Cannabis Commercialization With Self-harm Injury Rates, With Recreational Dispensary Categories Disaggregated by THC Dosage-Related Restrictions, by Age Group and Gender, 2003-2017 **eFigure 20.** Adjusted Associations of Medical and Recreational Cannabis Commercialization With Assault Injury Rates, With Medical and Recreational Policy Categories Collapsed, by Age Group and Gender, 2003-2017 **eFigure 21.** Adjusted Associations of Medical and Recreational Cannabis Commercialization With Self-harm Injury Rates, With Medical and Recreational Policy Categories Collapsed, by Age Group and Gender, 2003-2017 **eFigure 22.** Adjusted Associations of Naloxone Overdose Prevention Laws as a Negative Control Exposure With Assault Injury Rates, by Age Group and Gender, 2003-2017 **eFigure 23.** Adjusted Associations of Naloxone Overdose Prevention Laws as a Negative Control Exposure With Self-harm Injury Rates, by Age Group and Gender, 2003-2017 **eFigure 24.** Adjusted Associations of Hypothetical, Randomly Generated Law Changes as a Negative Control Exposure With Assault Injury Rates, by Age Group and Gender, 2003-2017 **eFigure 25.** Adjusted Associations of Hypothetical, Randomly Generated Law Changes as a Negative Control Exposure With Self-harm Injury Rates, by Age Group and Gender, 2003-2017 **eFigure 26.** Adjusted Associations of Medical and Recreational Cannabis Commercialization With Assault Injury Rates, With Additional Adjustment for Linear State-Specific Time Trends, by Age Group and Gender, 2003-2017 **eFigure 27.** Adjusted Associations of Medical and Recreational Cannabis Commercialization With Self-harm Injury Rates, With Additional Adjustment for Linear State-Specific Time Trends, by Age Group and Gender, 2003-2017 **eFigure 28.** Adjusted Associations of Medical and Recreational Cannabis Commercialization With Assault Injury Rates, for States With Cannabis Legalization, by Age Group and Gender, 2003-2017 **eFigure 29.** Adjusted Associations of Medical and Recreational Cannabis Commercialization With Self-harm Injury Rates, for States With Cannabis Legalization, by Age Group and Gender, 2003-2017
