# Error in End Matter

**DOI:** 10.1001/jamanetworkopen.2021.9962

**Published:** 2021-04-16

In the Original Investigation titled “Evaluation of State Cannabis Laws and Rates of Self-harm and Assault,”^1^ published March 18, 2021, there was an omission in the end matter. Following the Conflict of Interest Disclosures, sections listing Funding/Support, Role of the Funder/Sponsor, Disclaimer, and Additional Contributions should have been included. This article has been corrected.^1^
