# Supplementary material for: Evaluation of State Cannabis Laws and Rates of Self-harm and Assault
Source: JAMA Netw Open. 2021 Mar 18;4(3):e211955. doi: 10.1001/jamanetworkopen.2021.1955 (PMC7974641; doi:10.1001/jamanetworkopen.2021.1955)
Supplement: Supplement. — eMethods. Power Calculations eTable 1. Covariate Data Sources and Information eTable 2. International Classification of Diseases Clinical Modification (ICD-CM) 9th and 10th Revision Cause of Injury Codes Used to Identify and Classify Self-harm and Assault Injuries eTable 3. Characteristics of Study State-Months Overall and by Policy Type eTable 4. Beneficiary-Months of Observation and Baseline Self-harm and Assault Injury Rates for Age and Gender Subgroup Analyses eFigure 1. Adjusted Associations of Medical and Recreational Cannabis Commercialization With Self-harm and Assault Injury Rates, Using Unique Claims as the Denominator for Outcome Rates, for the Overall Study Population, 2003-2017 eFigure 2. Adjusted Associations of Medical and Recreational Cannabis Commercialization With Self-harm and Assault Injury Rates, Using Active Members as the Denominator for Outcome Rates, for the Overall Study Population, 2003-2017 eFigure 3. Adjusted Associations of Medical and Recreational Cannabis Commercialization With Self-harm and Assault Injury Rates, Adjusted for Firearm Availability, Without the District of Columbia, for the Overall Study Population, 2003-2017 eFigure 4. Adjusted Associations of Medical and Recreational Cannabis Commercialization With Self-harm and Assault Injury Rates, With Recreational Dispensary Categories Disaggregated by THC Dosage-Related Restrictions, for the Overall Study Population, 2003-2017 eFigure 5. Adjusted Associations of Medical and Recreational Cannabis Commercialization With Self-harm and Assault Injury Rates, With Medical and Recreational Policy Categories Collapsed, for the Overall Study Population, 2003-2017 eFigure 6. Adjusted Associations of Medical and Recreational Cannabis Commercialization With the All-Claims Rate as a Negative Control Outcome, for the Overall Study Population, 2003-2017 eFigure 7. Adjusted Associations of Naloxone Overdose Prevention Laws as a Negative Control Exposure With Self-harm and Assault Injury Rates, f [file jamanetwopen-e211955-s001.pdf]

Supplementary Online Content

Matthay EC, Kiang MV, Elser H, Schmidt L, Humphreys K. Evaluation of state cannabis laws and rates of self-harm and assault. *JAMA Netw Open*. 2021;4(3):e211955. doi:10.1001/jamanetworkopen.2021.1955

eMethods. Power Calculations

eTable 1. Covariate Data Sources and Information

eTable 2. International Classification of Diseases Clinical Modification (ICD-CM) 9th and 10th Revision Cause of Injury Codes Used to Identify and Classify Self-harm and Assault Injuries

eTable 3. Characteristics of Study State-Months Overall and by Policy Type

eTable 4. Beneficiary-Months of Observation and Baseline Self-harm and Assault Injury Rates for Age and Gender Subgroup Analyses

eFigure 1. Adjusted Associations of Medical and Recreational Cannabis Commercialization With Self-harm and Assault Injury Rates, Using Unique Claims as the Denominator for Outcome Rates, for the Overall Study Population, 2003-2017

eFigure 2. Adjusted Associations of Medical and Recreational Cannabis Commercialization With Self-harm and Assault Injury Rates, Using Active Members as the Denominator for Outcome Rates, for the Overall Study Population, 2003-2017

eFigure 3. Adjusted Associations of Medical and Recreational Cannabis Commercialization With Self-harm and Assault Injury Rates, Adjusted for Firearm Availability, Without the District of Columbia, for the Overall Study Population, 2003-2017

eFigure 4. Adjusted Associations of Medical and Recreational Cannabis Commercialization With Self-harm and Assault Injury Rates, With Recreational Dispensary Categories Disaggregated by THC Dosage-Related Restrictions, for the Overall Study Population, 2003-2017

eFigure 5. Adjusted Associations of Medical and Recreational Cannabis Commercialization With Self-harm and Assault Injury Rates, With Medical and Recreational Policy Categories Collapsed, for the Overall Study Population, 2003-2017

eFigure 6. Adjusted Associations of Medical and Recreational Cannabis Commercialization With the All-Claims Rate as a Negative Control Outcome, for the Overall Study Population, 2003-2017

eFigure 7. Adjusted Associations of Naloxone Overdose Prevention Laws as a Negative Control Exposure With Self-harm and Assault Injury Rates, for the Overall Study Population, 2003-2017

eFigure 8. Adjusted Associations of Hypothetical, Randomly Generated Law Changes as a Negative Control Exposure With Self-harm and Assault Injury Rates, for the Overall Study Population, 2003-2017

eFigure 9. Adjusted Associations of Medical and Recreational Cannabis Commercialization With Self-harm and Assault Injury Rates, With Additional Adjustment for Linear State-Specific Time Trends, for the Overall Study Population, 2003-2017

eFigure 10. Adjusted Associations of Medical and Recreational Cannabis Commercialization With Self-harm and Assault Injury Rates, for States With Cannabis Legalization, for the Overall Study Population, 2003-2017

eFigure 11. Adjusted Associations of Recreational (Versus Medical) Cannabis Legalization and Commercialization (Dispensaries Versus Home-Grown Only) With Self-harm and Assault Injury Rates, for States With Cannabis Legalization, for the Overall Study Population, 2003-2017

eFigure 12. Adjusted Associations of Medical and Recreational Cannabis Commercialization With Self-harm Injury Rates, Using Unique Claims as the Denominator for Outcome Rates, by Age Group and Gender, 2003-2017

eFigure 13. Adjusted Associations of Medical and Recreational Cannabis Commercialization With Self-harm Injury Rates, Using Unique Claims as the Denominator for Outcome Rates, by Age Group and Gender, 2003-2017

eFigure 14. Adjusted Associations of Medical and Recreational Cannabis Commercialization With Assault Injury Rates, Using Active Members as the Denominator for Outcome Rates, by Age Group and Gender, 2003-2017

© 2021 Matthay EC et al. *JAMA Network Open*.

eFigure 15. Adjusted Associations of Medical and Recreational Cannabis Commercialization With Self-harm Injury Rates, Using Active Members as the Denominator for Outcome Rates, by Age Group and Gender, 2003-2017

eFigure 16. Adjusted Associations of Medical and Recreational Cannabis Commercialization With Assault Injury Rates, Adjusted for Firearm Availability, Without the District of Columbia, by Age Group and Gender, 2003-2017

eFigure 17. Adjusted Associations of Medical and Recreational Cannabis Commercialization With Self-harm Injury Rates, Adjusted for Firearm Availability, Without the District of Columbia, by Age Group and Gender, 2003-2017

eFigure 18. Adjusted Associations of Medical and Recreational Cannabis Commercialization With Assault Injury Rates, With Recreational Dispensary Categories Disaggregated by THC Dosage-Related Restrictions, by Age Group and Gender, 2003-2017

eFigure 19. Adjusted Associations of Medical and Recreational Cannabis Commercialization With Self-harm Injury Rates, With Recreational Dispensary Categories Disaggregated by THC Dosage-Related Restrictions, by Age Group and Gender, 2003-2017

eFigure 20. Adjusted Associations of Medical and Recreational Cannabis Commercialization With Assault Injury Rates, With Medical and Recreational Policy Categories Collapsed, by Age Group and Gender, 2003-2017

eFigure 21. Adjusted Associations of Medical and Recreational Cannabis Commercialization With Self-harm Injury Rates, With Medical and Recreational Policy Categories Collapsed, by Age Group and Gender, 2003-2017

eFigure 22. Adjusted Associations of Naloxone Overdose Prevention Laws as a Negative Control Exposure With Assault Injury Rates, by Age Group and Gender, 2003-2017

eFigure 23. Adjusted Associations of Naloxone Overdose Prevention Laws as a Negative Control Exposure With Self-harm Injury Rates, by Age Group and Gender, 2003-2017

eFigure 24. Adjusted Associations of Hypothetical, Randomly Generated Law Changes as a Negative Control Exposure With Assault Injury Rates, by Age Group and Gender, 2003-2017

eFigure 25. Adjusted Associations of Hypothetical, Randomly Generated Law Changes as a Negative Control Exposure With Self-harm Injury Rates, by Age Group and Gender, 2003-2017

eFigure 26. Adjusted Associations of Medical and Recreational Cannabis Commercialization With Assault Injury Rates, With Additional Adjustment for Linear State-Specific Time Trends, by Age Group and Gender, 2003-2017

eFigure 27. Adjusted Associations of Medical and Recreational Cannabis Commercialization With Self-harm Injury Rates, With Additional Adjustment for Linear State-Specific Time Trends, by Age Group and Gender, 2003-2017

eFigure 28. Adjusted Associations of Medical and Recreational Cannabis Commercialization With Assault Injury Rates, for States With Cannabis Legalization, by Age Group and Gender, 2003-2017

eFigure 29. Adjusted Associations of Medical and Recreational Cannabis Commercialization With Self-harm Injury Rates, for States With Cannabis Legalization, by Age Group and Gender, 2003-2017

This supplementary material has been provided by the authors to give readers additional information about their work.

© 2021 Matthay EC et al. *JAMA Network Open*.

Supplemental Methods

Power calculations

To confirm that our study had sufficient statistical power, we conducted a power analysis. Using the observed baseline rates of self-harm and assault with a negative binomial distribution, the main exposure measure, and a two-sided test with alpha 0.05, the analysis had 80% power to detect a minimum 5.1% increase in assault and 5.3% increase in self-harm for the rarest commercialization category.

© 2021 Matthay EC et al. *JAMA Network Open*.

eTable 1: Covariate data sources and information

Covariates	Source	Time scale	Additional information
Percent non-Hispanic Asian, percent non-Hispanic Black, percent non-Hispanic White, percent Hispanic	US Census (2002-2009); American Community Survey 1-year estimates (2010-2017)	Annual	
Percent in poverty, median income,	Small Area Income and Poverty Estimates Program (SAIPE) (2002-2017)	Annual	
Percent unemployed	Bureau of Labor Statistics Local Area Unemployment Files	Monthly	
Percent veterans, percent renters	US Census (2000); American Community Survey 1-year estimates (2010-2017)	Annual	Linear interpolation assumed for 2000-2010.
Violent crime rate	FBI Uniform Crime Reports	Annual	
Percent females aged 15-29 years, percent females aged 30-44 years, percent males aged 15-29 years, percent males aged 30-44 years	Optum Clinformatics Data Mart	Monthly	
Alcohol policy stringency	Blanchette JG, Lira MC, Heeren TC, Naimi TS. Alcohol Policies in U.S. States, 1999–2018. *J Stud Alcohol Drugs*. 2020;81(1):58-67. doi:10.15288/jsad.2020.81.58	Annual	
Opioid overdose mortality rate	Age-standardized mortality rates by state and month from National Center for Health Statistics mortality data and corresponding population estimates from the US Census.	Monthly	
Overall claims rate	Optum Clinformatics Data Mart	Monthly	
Indicator for the October 1, 2015 shift from the 9th to 10th ICD revision	None	Before versus after October 1, 2015	
Firearm access (percent of suicides completed with a firearm)	CDC WISQARS Fatal Injury Reports	Annual	Measure unavailable for the District of Columbia.

© 2021 Matthay EC et al. *JAMA Network Open*.

eTable 2: International Classification of Diseases Clinical Modification (ICD-CM) 9th and 10th revision external cause of injury codes used to identify and classify self-harm and assault injuries

	Self-harm injury	Assault injury
ICD-9-CM codes	E950, E9500, E9501, E9502, E9503, E9504, E9505, E9506, E9507, E9508, E9509, E951, E9510, E9511, E9518, E952, E9520, E9521, E9528, E9529, E953, E9530, E9531, E9538, E9539, E954, E955, E9550, E9551, E9552, E9553, E9554, E9555, E9556, E9557, E9559, E956, E957, E9570, E9571, E9572, E9579, E958, E9580, E9581, E9582, E9583, E9584, E9585, E9586, E9587, E9588, E9589, E959	E960, E9600, E9601, E961, E962, E9620, E9621, E9622, E9629, E963, E964, E965, E9650, E9651, E9652, E9653, E9654, E9655, E9656, E9657, E9658, E9659, E966, E967, E9670, E9671, E9672, E9673, E9674, E9675, E9676, E9677, E9678, E9679, E968, E9680, E9681, E9682, E9683, E9684, E9685, E9686, E9687, E9688, E9689, E969, E970, E971, E972, E973, E974, E975, E976, E977
ICD-10-CM codes	X71, X710, X710XXA, X711, X711XXA, X712, X712XXA, X713, X713XXA, X718, X718XXA, X719, X719XXA, X72, X72XXXA, X73, X730, X730XXA, X731, X731XXA, X732, X732XXA, X738, X738XXA, X739, X739XXA, X74, X740, X7401, X7401XA, X7402, X7402XA, X7409, X7409XA, X748, X748XXA, X749, X749XXA, X75, X75XXXA, X76, X76XXXA, X77, X770, X770XXA, X771, X771XXA, X772, X772XXA, X773, X773XXA, X778, X778XXA, X779, X779XXA, X78, X780, X780XXA, X781, X781XXA, X782, X782XXA, X788, X788XXA, X789, X789XXA, X79, X79XXXA, X80, X80XXXA, X81, X810, X810XXA, X811, X811XXA, X818, X818XXA, X82, X820, X820XXA, X821, X821XXA, X822, X822XXA, X828, X828XXA, X83, X830, X830XXA, X831, X831XXA, X832, X832XXA, X838, X838XXA, T360X2, T360X2A, T361X2, T361X2A, T362X2, T362X2A, T363X2, T363X2A, T364X2, T364X2A, T365X2, T365X2A, T366X2, T366X2A, T367X2, T367X2A, T368X2, T368X2A, T3692XA, T370X2, T370X2A, T371X2, T371X2A, T372X2, T372X2A, T373X2, T373X2A, T374X2, T374X2A, T375X2, T375X2A, T378X2, T378X2A, T3792XA, T380X2, T380X2A, T381X2, T381X2A, T382X2, T382X2A, T383X2, T383X2A, T384X2, T384X2A, T385X2, T385X2A, T386X2, T386X2A, T387X2, T387X2A, T38802, T38802A, T38812, T38812A, T38892, T38892A, T38902, T38902A, T38992, T38992A, T39012, T39012A, T39092, T39092A, T391X2, T391X2A, T392X2, T392X2A, T39312, T39312A, T39392, T39392A, T394X2, T394X2A, T398X2, T398X2A, T3992XA, T400X2, T400X2A, T401X2, T401X2A, T402X2, T402X2A, T403X2, T403X2A, T404X2, T404X2A, T405X2, T405X2A, T40602, T40602A,	X92, X920, X920XXA, X921, X921XXA, X922, X922XXA, X923, X923XXA, X928, X928XXA, X929, X929XXA, X93, X93XXXA, X94, X940, X940XXA, X941, X941XXA, X942, X942XXA, X948, X948XXA, X949, X949XXA, X95, X950, X9501, X9501XA, X9502, X9502XA, X9509, X9509XA, X958, X958XXA, X959, X959XXA, X96, X960, X960XXA, X961, X961XXA, X962, X962XXA, X963, X963XXA, X964, X964XXA, X968, X968XXA, X969, X969XXA, X97, X97XXXA, X98, X980, X980XXA, X981, X981XXA, X982, X982XXA, X983, X983XXA, X988, X988XXA, X989, X989XXA, X99, X990, X990XXA, X991, X991XXA, X992, X992XXA, X998, X998XXA, X999, X999XXA, Y00, Y00XXXA, Y01, Y01XXXA, Y02, Y020, Y020XXA, Y021, Y021XXA, Y028, Y028XXA, Y03, Y030, Y030XXA, Y038, Y038XXA, Y04, Y040, Y040XXA, Y041, Y041XXA, Y042, Y042XXA, Y048, Y048XXA, Y07, Y070, Y0701, Y0702, Y0703, Y0704, Y071, Y0711, Y0712, Y0713, Y0714, Y074, Y0741, Y07410, Y07411, Y0742, Y07420, Y07421, Y0743, Y07430, Y07432, Y07433, Y07434, Y07435, Y07436, Y0749, Y07490, Y07491, Y07499, Y075, Y0750, Y0751, Y07510, Y07511, Y07512, Y07513, Y07519, Y0752, Y07521, Y07528, Y07529, Y0753, Y0759, Y079, Y08, Y080, Y0801, Y0801XA, Y0802, Y0802XA, Y0809, Y0809XA, Y088, Y0881, Y0881XA, Y0889, Y0889XA, Y09, Y35, Y350, Y3500, Y35001, Y35001A, Y35002, Y35002A, Y35003, Y35003A, Y3501, Y35011, Y35011A, Y35012, Y35012A, Y35013, Y35013A, Y3502, Y35021, Y35021A, Y35022, Y35022A, Y35023, Y35023A, Y3503, Y35031, Y35031A, Y35032, Y35032A, Y35033, Y35033A, Y3504, Y35041, Y35041A, Y35042, Y35042A, Y35043, Y35043A, Y3509, Y35091, Y35091A,

© 2021 Matthay EC et al. *JAMA Network Open*.

	T40692, T40692A, T407X2, T407X2A, T408X2, T408X2A, T40902, T40902A, T40992, T40992A, T410X2, T410X2A, T411X2, T411X2A, T41202, T41202A, T41292, T41292A, T413X2, T413X2A, T4142XA, T415X2, T415X2A, T420X2, T420X2A, T421X2, T421X2A, T422X2, T422X2A, T423X2, T423X2A, T424X2, T424X2A, T425X2, T425X2A, T426X2, T426X2A, T4272XA, T428X2, T428X2A, T43012, T43012A, T43022, T43022A, T431X2, T431X2A, T43202, T43202A, T43212, T43212A, T43222, T43222A, T43292, T43292A, T433X2, T433X2A, T434X2, T434X2A, T43502, T43502A, T43592, T43592A, T43602, T43602A, T43612, T43612A, T43622, T43622A, T43632, T43632A, T43692, T43692A, T438X2, T438X2A, T4392XA, T440X2, T440X2A, T441X2, T441X2A, T442X2, T442X2A, T443X2, T443X2A, T444X2, T444X2A, T445X2, T445X2A, T446X2, T446X2A, T447X2, T447X2A, T448X2, T448X2A, T44902, T44902A, T44992, T44992A, T450X2, T450X2A, T451X2, T451X2A, T452X2, T452X2A, T453X2, T453X2A, T454X2, T454X2A, T45512, T45512A, T45522, T45522A, T45602, T45602A, T45612, T45612A, T45622, T45622A, T45692, T45692A, T457X2, T457X2A, T458X2, T458X2A, T4592XA, T460X2, T460X2A, T461X2, T461X2A, T462X2, T462X2A, T463X2, T463X2A, T464X2, T464X2A, T465X2, T465X2A, T466X2, T466X2A, T467X2, T467X2A, T468X2, T468X2A, T46902, T46902A, T46992, T46992A, T470X2, T470X2A, T471X2, T471X2A, T472X2, T472X2A, T473X2, T473X2A, T474X2, T474X2A, T475X2, T475X2A, T476X2, T476X2A, T477X2, T477X2A, T478X2, T478X2A, T4792XA, T480X2, T480X2A, T481X2, T481X2A, T48202, T48202A, T48292, T48292A, T483X2, T483X2A, T484X2, T484X2A, T485X2, T485X2A, T486X2, T486X2A, T48902, T48902A, T48992, T48992A, T490X2, T490X2A, T491X2, T491X2A, T492X2, T492X2A, T493X2, T493X2A, T494X2, T494X2A, T495X2, T495X2A, T496X2, T496X2A, T497X2, T497X2A, T498X2, T498X2A, T4992XA, T500X2, T500X2A, T501X2, T501X2A, T502X2, T502X2A, T503X2, T503X2A, T504X2, T504X2A, T505X2, T505X2A, T506X2, T506X2A, T507X2, T507X2A, T508X2, T508X2A, T50902, T50902A,	Y35092, Y35092A, Y35093, Y35093A, Y351, Y3510, Y35101, Y35101A, Y35102, Y35102A, Y35103, Y35103A, Y3511, Y35111, Y35111A, Y35112, Y35112A, Y35113, Y35113A, Y3512, Y35121, Y35121A, Y35122, Y35122A, Y35123, Y35123A, Y3519, Y35191, Y35191A, Y35192, Y35192A, Y35193, Y35193A, Y352, Y3520, Y35201, Y35201A, Y35202, Y35202A, Y35203, Y35203A, Y3521, Y35211, Y35211A, Y35212, Y35212A, Y35213, Y35213A, Y3529, Y35291, Y35291A, Y35292, Y35292A, Y35293, Y35293A, Y353, Y3530, Y35301, Y35301A, Y35302, Y35302A, Y35303, Y35303A, Y3531, Y35311, Y35311A, Y35312, Y35312A, Y35313, Y35313A, Y3539, Y35391, Y35391A, Y35392, Y35392A, Y35393, Y35393A, Y354, Y3540, Y35401, Y35401A, Y35402, Y35402A, Y35403, Y35403A, Y3541, Y35411, Y35411A, Y35412, Y35412A, Y35413, Y35413A, Y3549, Y35491, Y35491A, Y35492, Y35492A, Y35493, Y35493A, Y358, Y3581, Y35811, Y35811A, Y35812, Y35812A, Y35813, Y35813A, Y3589, Y35891, Y35891A, Y35892, Y35892A, Y35893, Y35893A, Y359, Y3591, Y3591XA, Y3592, Y3592XA, Y3593, Y3593XA, T360X3, T360X3A, T361X3, T361X3A, T362X3, T362X3A, T363X3, T363X3A, T364X3, T364X3A, T365X3, T365X3A, T366X3, T366X3A, T367X3, T367X3A, T368X3, T368X3A, T3693XA, T370X3, T370X3A, T371X3, T371X3A, T372X3, T372X3A, T373X3, T373X3A, T374X3, T374X3A, T375X3, T375X3A, T378X3, T378X3A, T3793XA, T380X3, T380X3A, T381X3, T381X3A, T382X3, T382X3A, T383X3, T383X3A, T384X3, T384X3A, T385X3, T385X3A, T386X3, T386X3A, T387X3, T387X3A, T38803, T38803A, T38813, T38813A, T38893, T38893A, T38903, T38903A, T38993, T38993A, T39013, T39013A, T39093, T39093A, T391X3, T391X3A, T392X3, T392X3A, T39313, T39313A, T39393, T39393A, T394X3, T394X3A, T398X3, T398X3A, T3993XA, T400X3, T400X3A, T401X3, T401X3A, T402X3, T402X3A, T403X3, T403X3A, T404X3, T404X3A, T405X3, T405X3A, T40603, T40603A, T40693, T40693A, T407X3, T407X3A, T408X3, T408X3A, T40903, T40903A, T40993, T40993A, T410X3, T410X3A, T411X3, T411X3A, T41203, T41203A, T41293, T41293A,

	T50992, T50992A, T50A12, T50A12A, T50A22, T50A22A, T50A92, T50A92A, T50B12, T50B12A, T50B92, T50B92A, T50Z12, T50Z12A, T50Z92, T50Z92A, T510X2, T510X2A, T511X2, T511X2A, T512X2, T512X2A, T513X2, T513X2A, T518X2, T518X2A, T5192XA, T520X2, T520X2A, T521X2, T521X2A, T522X2, T522X2A, T523X2, T523X2A, T524X2, T524X2A, T528X2, T528X2A, T5292XA, T530X2, T530X2A, T531X2, T531X2A, T532X2, T532X2A, T533X2, T533X2A, T534X2, T534X2A, T535X2, T535X2A, T536X2, T536X2A, T537X2, T537X2A, T5392XA, T540X2, T540X2A, T541X2, T541X2A, T542X2, T542X2A, T543X2, T543X2A, T5492XA, T550X2, T550X2A, T551X2, T551X2A, T560X2, T560X2A, T561X2, T561X2A, T562X2, T562X2A, T563X2, T563X2A, T564X2, T564X2A, T565X2, T565X2A, T566X2, T566X2A, T567X2, T567X2A, T56812, T56812A, T56892, T56892A, T5692XA, T570X2, T570X2A, T571X2, T571X2A, T572X2, T572X2A, T573X2, T573X2A, T578X2, T578X2A, T5792XA, T5802XA, T5812XA, T582X2, T582X2A, T588X2, T588X2A, T5892XA, T590X2, T590X2A, T591X2, T591X2A, T592X2, T592X2A, T593X2, T593X2A, T594X2, T594X2A, T595X2, T595X2A, T596X2, T596X2A, T597X2, T597X2A, T59812, T59812A, T59892, T59892A, T5992XA, T600X2, T600X2A, T601X2, T601X2A, T602X2, T602X2A, T603X2, T603X2A, T604X2, T604X2A, T608X2, T608X2A, T6092XA, T6102XA, T6112XA, T61772, T61772A, T61782, T61782A, T618X2, T618X2A, T6192XA, T620X2, T620X2A, T621X2, T621X2A, T622X2, T622X2A, T628X2, T628X2A, T6292XA, T63002, T63002A, T63012, T63012A, T63022, T63022A, T63032, T63032A, T63042, T63042A, T63062, T63062A, T63072, T63072A, T63082, T63082A, T63092, T63092A, T63112, T63112A, T63122, T63122A, T63192, T63192A, T632X2, T632X2A, T63302, T63302A, T63312, T63312A, T63322, T63322A, T63332, T63332A, T63392, T63392A, T63412, T63412A, T63422, T63422A, T63432, T63432A, T63442, T63442A, T63452, T63452A, T63462, T63462A, T63482, T63482A, T63512, T63512A, T63592, T63592A, T63612, T63612A, T63622, T63622A, T63632, T63632A, T63692, T63692A, T63812,	T413X3, T413X3A, T4143XA, T415X3, T415X3A, T420X3, T420X3A, T421X3, T421X3A, T422X3, T422X3A, T423X3, T423X3A, T424X3, T424X3A, T425X3, T425X3A, T426X3, T426X3A, T4273XA, T428X3, T428X3A, T43013, T43013A, T43023, T43023A, T431X3, T431X3A, T43203, T43203A, T43213, T43213A, T43223, T43223A, T43293, T43293A, T433X3, T433X3A, T434X3, T434X3A, T43503, T43503A, T43593, T43593A, T43603, T43603A, T43613, T43613A, T43623, T43623A, T43633, T43633A, T43693, T43693A, T438X3, T438X3A, T4393XA, T440X3, T440X3A, T441X3, T441X3A, T442X3, T442X3A, T443X3, T443X3A, T444X3, T444X3A, T445X3, T445X3A, T446X3, T446X3A, T447X3, T447X3A, T448X3, T448X3A, T44903, T44903A, T44993, T44993A, T450X3, T450X3A, T451X3, T451X3A, T452X3, T452X3A, T453X3, T453X3A, T454X3, T454X3A, T45513, T45513A, T45523, T45523A, T45603, T45603A, T45613, T45613A, T45623, T45623A, T45693, T45693A, T457X3, T457X3A, T458X3, T458X3A, T4593XA, T460X3, T460X3A, T461X3, T461X3A, T462X3, T462X3A, T463X3, T463X3A, T464X3, T464X3A, T465X3, T465X3A, T466X3, T466X3A, T467X3, T467X3A, T468X3, T468X3A, T46903, T46903A, T46993, T46993A, T470X3, T470X3A, T471X3, T471X3A, T472X3, T472X3A, T473X3, T473X3A, T474X3, T474X3A, T475X3, T475X3A, T476X3, T476X3A, T477X3, T477X3A, T478X3, T478X3A, T4793XA, T480X3, T480X3A, T481X3, T481X3A, T48203, T48203A, T48293, T48293A, T483X3, T483X3A, T484X3, T484X3A, T485X3, T485X3A, T486X3, T486X3A, T48903, T48903A, T48993, T48993A, T490X3, T490X3A, T491X3, T491X3A, T492X3, T492X3A, T493X3, T493X3A, T494X3, T494X3A, T495X3, T495X3A, T496X3, T496X3A, T497X3, T497X3A, T498X3, T498X3A, T4993XA, T500X3, T500X3A, T501X3, T501X3A, T502X3, T502X3A, T503X3, T503X3A, T504X3, T504X3A, T505X3, T505X3A, T506X3, T506X3A, T507X3, T507X3A, T508X3, T508X3A, T50903, T50903A, T50993, T50993A, T50A13, T50A13A, T50A23, T50A23A, T50A93, T50A93A, T50B13, T50B13A, T50B93, T50B93A, T50Z13, T50Z13A, T50Z93, T50Z93A, T510X3, T510X3A,

© 2021 Matthay EC et al. *JAMA Network Open*.

	T63812A, T63822, T63822A, T63832, T63832A, T63892, T63892A, T6392XA, T63712, T63712A, T63792, T63792A, T6402XA, T6482XA, T650X2, T650X2A, T651X2, T651X2A, T65212, T65212A, T65222, T65222A, T65292, T65292A, T653X2, T653X2A, T654X2, T654X2A, T655X2, T655X2A, T656X2, T656X2A, T65812, T65812A, T65822, T65822A, T65832, T65832A, T65892, T65892A, T6592XA, T71112, T71112A, T71122, T71122A, T71132, T71132A, T71152, T71152A, T71162, T71162A, T71192, T71192A, T71222, T71222A, T71232, T71232A	T511X3, T511X3A, T512X3, T512X3A, T513X3, T513X3A, T518X3, T518X3A, T5193XA, T520X3, T520X3A, T521X3, T521X3A, T522X3, T522X3A, T523X3, T523X3A, T524X3, T524X3A, T528X3, T528X3A, T5293XA, T530X3, T530X3A, T531X3, T531X3A, T532X3, T532X3A, T533X3, T533X3A, T534X3, T534X3A, T535X3, T535X3A, T536X3, T536X3A, T537X3, T537X3A, T5393XA, T540X3, T540X3A, T541X3, T541X3A, T542X3, T542X3A, T543X3, T543X3A, T5493XA, T550X3, T550X3A, T551X3, T551X3A, T560X3, T560X3A, T561X3, T561X3A, T562X3, T562X3A, T563X3, T563X3A, T564X3, T564X3A, T565X3, T565X3A, T566X3, T566X3A, T567X3, T567X3A, T56813, T56813A, T56893, T56893A, T5693XA, T570X3, T570X3A, T571X3, T571X3A, T572X3, T572X3A, T573X3, T573X3A, T578X3, T578X3A, T5793XA, T5803XA, T5813XA, T582X3, T582X3A, T588X3, T588X3A, T5893XA, T590X3, T590X3A, T591X3, T591X3A, T592X3, T592X3A, T593X3, T593X3A, T594X3, T594X3A, T595X3, T595X3A, T596X3, T596X3A, T597X3, T597X3A, T59813, T59813A, T59893, T59893A, T5993XA, T600X3, T600X3A, T601X3, T601X3A, T602X3, T602X3A, T603X3, T603X3A, T604X3, T604X3A, T608X3, T608X3A, T6093XA, T6103XA, T6113XA, T61773, T61773A, T61783, T61783A, T618X3, T618X3A, T6193XA, T620X3, T620X3A, T621X3, T621X3A, T622X3, T622X3A, T628X3, T628X3A, T6293XA, T63003, T63003A, T63013, T63013A, T63023, T63023A, T63033, T63033A, T63043, T63043A, T63063, T63063A, T63073, T63073A, T63083, T63083A, T63093, T63093A, T63113, T63113A, T63123, T63123A, T63193, T63193A, T632X3, T632X3A, T63303, T63303A, T63313, T63313A, T63323, T63323A, T63333, T63333A, T63393, T63393A, T63413, T63413A, T63423, T63423A, T63433, T63433A, T63443, T63443A, T63453, T63453A, T63463, T63463A, T63483, T63483A, T63513, T63513A, T63593, T63593A, T63613, T63613A, T63623, T63623A, T63633, T63633A, T63693, T63693A, T63813, T63813A, T63823, T63823A, T63833, T63833A, T63893, T63893A, T6393XA, T63713, T63713A, T63793, T63793A, T6403XA, T6483XA, T650X3, T650X3A, T651X3, T651X3A,

© 2021 Matthay EC et al. *JAMA Network Open*.

		T65213, T65213A, T65223, T65223A, T65293, T65293A, T653X3, T653X3A, T654X3, T654X3A, T655X3, T655X3A, T656X3, T656X3A, T65813, T65813A, T65823, T65823A, T65833, T65833A, T65893, T65893A, T6593XA, T71113, T71113A, T71123, T71123A, T71133, T71133A, T71143, T71143A, T71153, T71153A, T71163, T71163A, T71193, T71193A, T71223, T71223A, T71233, T71233A

© 2021 Matthay EC et al. *JAMA Network Open*.

Supplemental Results

© 2021 Matthay EC et al. *JAMA Network Open*.

eTable 3: Characteristics of study state-months overall and by policy type

Variable	Overall	No cannabis legalization policy	Medical, no dispensaries	Medical, dispensaries	Recreational, no dispensaries	Recreational, dispensaries
State-months (%)	9180 (100.0)	6144 (67.0)	1469 (16.2)	1259 (13.9)	125 (1.4)	138 (1.5)
States contributing any time at risk	51 (100)	43 (84)	28 (55)	23 (45)	8 (16)	5 (10)
Self-harm injury claim rate (per 100,000 persons, annualized [95% CI])	79.5 (79.0, 80.0)	75.4 (74.2, 76.6)	73.0 (72.7, 73.3)	95.1 (92.5, 97.7)	110.8 (109.4, 112.2)	162.8 (161.8, 163.8)
Assault injury claim rate (per 100,000 persons, annualized [95% CI])	93.9 (92.8, 95.0)	96.9 (95.5, 98.3)	92.5 (92.1, 92.9)	80.5 (78.1, 82.9)	106.5 (105.1, 107.9)	91.8 (91.1, 92.5)
% male aged 15-29 years	9.0	9.4	8.2	8.2	8.9	8.0
% female aged 15-29 years	8.9	9.3	8.3	7.9	8.9	7.6
% Asian	3.6	2.2	7.8	4.9	5.9	5.4
% Black	11.0	12.5	8.6	6.7	15.9	3.6
% Hispanic	10.2	8.3	9.6	18.5	14.3	15.6
% White	71.5	74.2	66.4	65.8	57.7	69.3
% in poverty	13.7	14.0	11.7	14.4	14.2	12.5
Median income ($)	50,048	47,171	55,500	54,526	66,023	62,217
% unemployed	6.0	5.8	5.9	7.1	6.3	5.4
% renters	33.7	32.4	35.8	36.2	44.0	37.4
% single-parent households	6.9	7.1	6.6	6.7	6.6	5.8
% veterans	10.4	10.6	11.3	9.1	8.2	9.7
% firearm suicides	52.1	54.9	45.7	46.5	47.7	50.5
Violent crime rate (per 100,000 persons, annual)	403.8	403.0	400.6	392.1	638.8	363.5
Alcohol Policy Score*	42.8	42.9	42.5	42.1	42.7	42.6
Overall claim rate (per 100,000 persons, annualized [95% CI])	802,417 (801,491, 803,343)	772,958 (771,857, 774,059)	842,175 (841,831, 842,519)	878,621 (876,427, 880,815)	897,350 (896,067, 898,633)	869,423 (868,729, 870,117)

* Range: 0 to 100; 0: least restrictive. 100: most restrictive.

© 2021 Matthay EC et al. *JAMA Network Open*.

eTable 4: Beneficiary-months of observation and baseline self-harm and assault injury rates for age and gender subgroup analyses

**Subgroup**	**Beneficiary-months of observation**	**Baseline self-harm injury claim rate (per 100,000 persons, annualized [95% CI])**	**Baseline assault injury claim rate (per 100,000 persons, annualized [95% CI])**
Women, under 21 years	267,114,523	125.0 (121.1 – 128.9)	65.9 (63.0 – 68.8)
Women, 21-39 years	306,409,253	100.5 (97.3 – 103.7)	100.2 (97.0 – 103.4)
Women, 40-64 years	425,770,567	90.6 (88.0 – 93.2)	51.0 (49.1 – 52.9)
Women, 65 years and older	237,543,284	43.3(40.9 – 45.7)	28.6 (26.6 – 30.6)
Men, under 21 years	278,632,987	52.9 (50.5 – 55.3)	128.4 (124.7 – 132.1)
Men, 21-39 years	299,574,900	65.7 (62.9 – 68.5)	178.4 (173.8 – 183.0)
Men, 40-64 years	412,363,604	53.1 (51.0 – 55.2)	72.6 (70.2 – 75.0)
Men, 65 years and older	183,075,491	65.9 (62.6 – 69.2)	40.4 (37.9 – 42.9)

Abbreviations: CI: confidence interval

© 2021 Matthay EC et al. *JAMA Network Open*.

eFigure 1: Adjusted associations of medical and recreational cannabis commercialization with self-harm and assault injury rates, using unique claims as the denominator for outcome rates, for the overall study population, 2003-2017

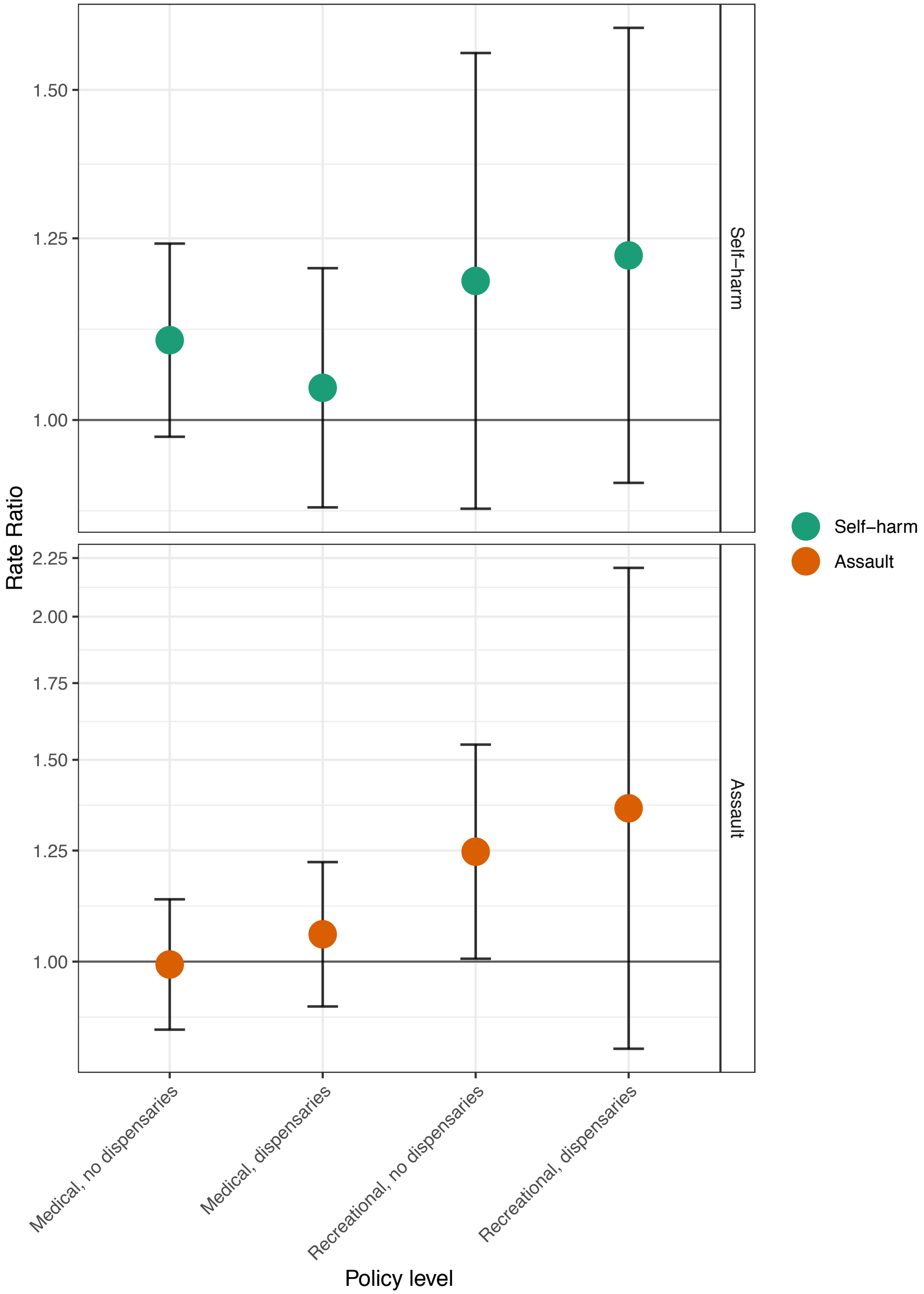

Legend: Points represent the estimated rate ratio for the association of each type of cannabis commercialization with self-harm or assault injuries, relative to state-months not adopting any medical or recreational cannabis legalization over the same period. Bars represent the corresponding 95% confidence intervals. The primary analysis used the number of insured beneficiaries in the given state and month as the denominator when calculating rates of self-harm and assault. In this sensitivity analysis, we used unique claims (the number of unique claims made per state-month) as the denominator, to account for changes in overall utilization over time.

© 2021 Matthay EC et al. *JAMA Network Open*.

eFigure 2: Adjusted associations of medical and recreational cannabis commercialization with self-harm and assault injury rates, using active members as the denominator for outcome rates, for the overall study population, 2003-2017

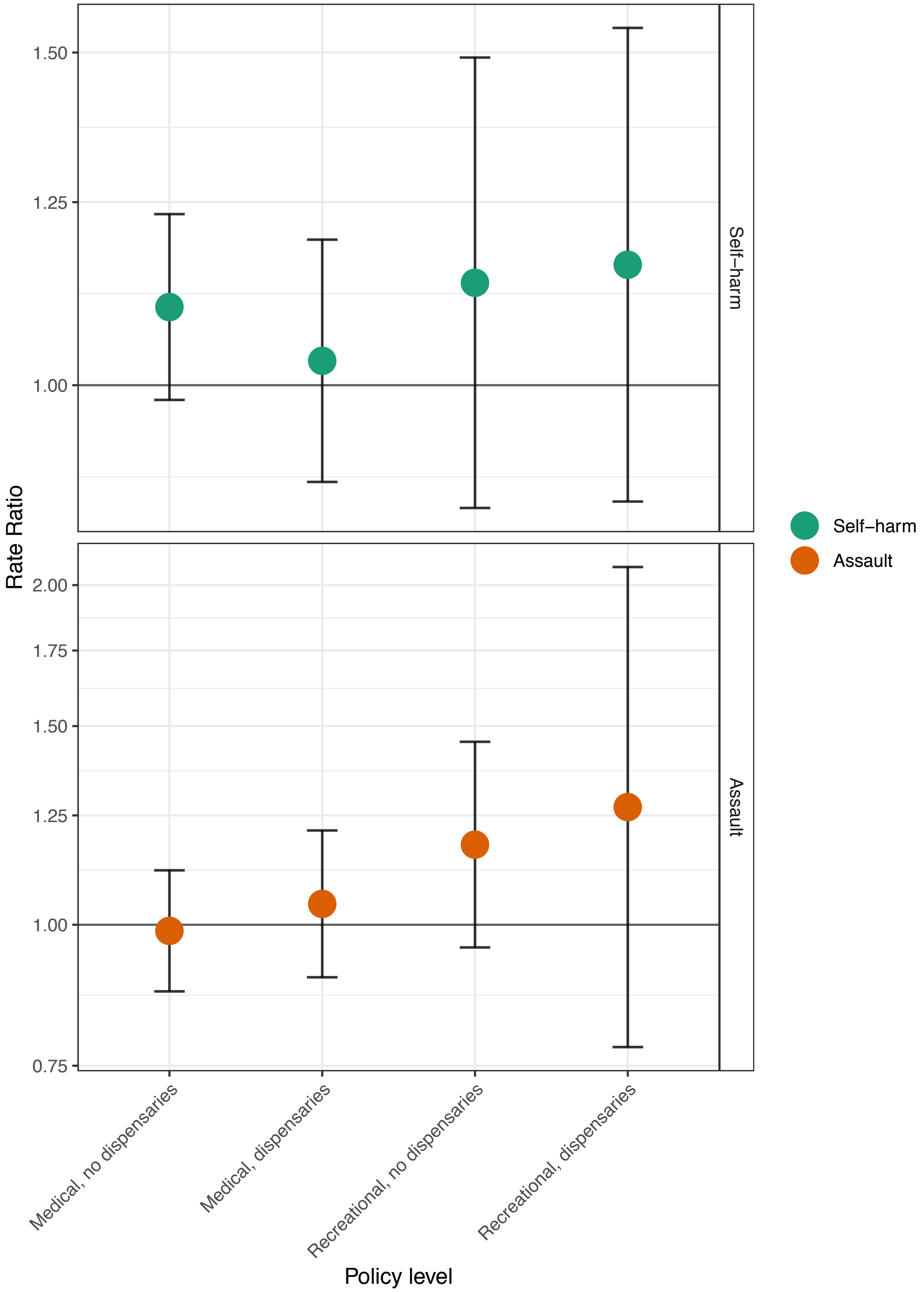

Legend: Points represent the estimated rate ratio for the association of each type of cannabis commercialization with self-harm or assault injuries, relative to state-months not adopting any medical or recreational cannabis legalization over the same period. Bars represent the corresponding 95% confidence intervals. The primary analysis used the number of insured beneficiaries in the given state and month as the denominator when calculating rates of self-harm and assault. In this sensitivity analysis, we used active members (the number of unique members who filed at least one claim per state month), because this approach is more robust to high-utilizing members.

© 2021 Matthay EC et al. *JAMA Network Open*.

eFigure 3: Adjusted associations of medical and recreational cannabis commercialization with self-harm and assault injury rates, adjusted for firearm availability, without the District of Columbia, for the overall study population, 2003-2017

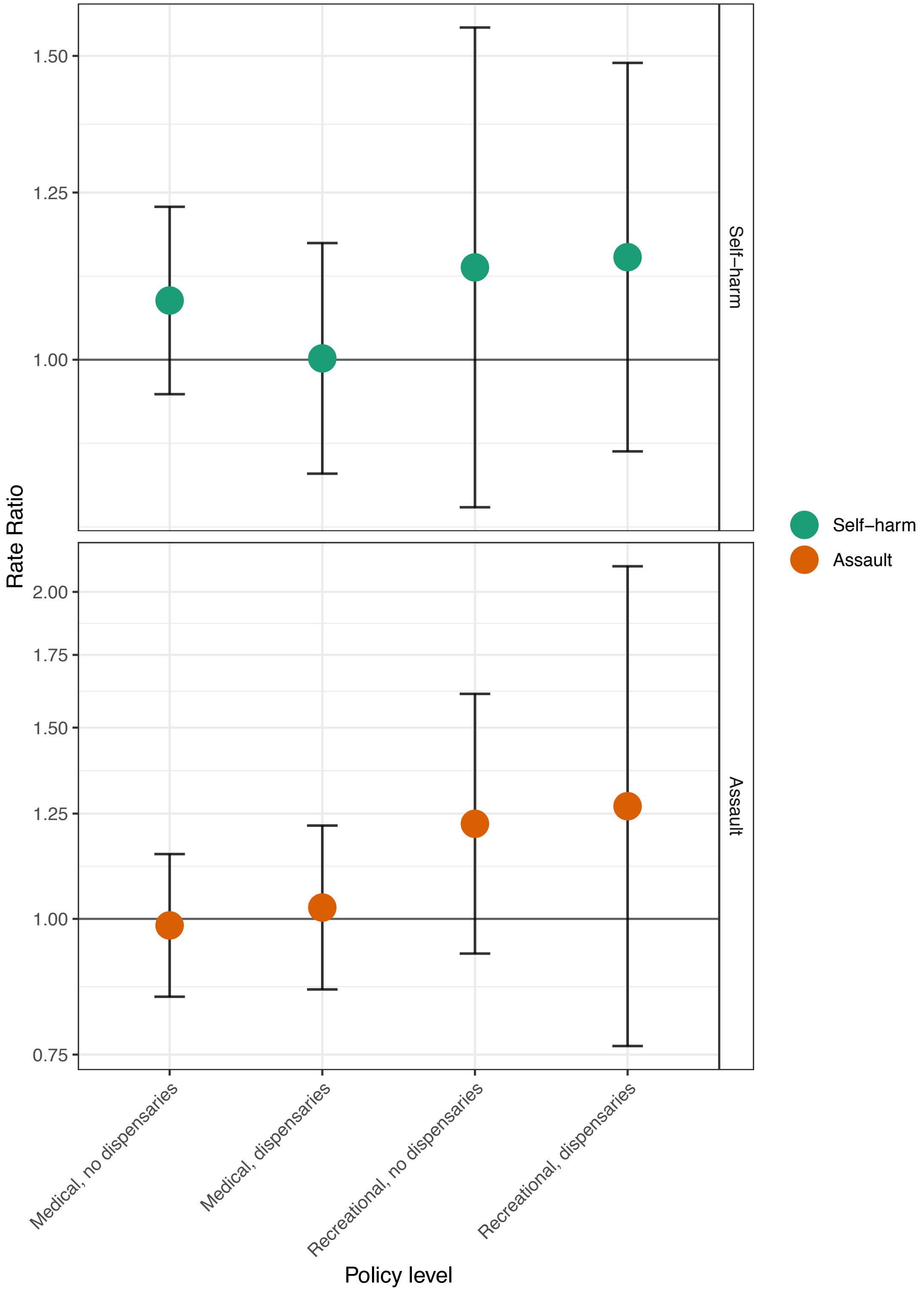

Legend: Points represent the estimated rate ratio for the association of each type of cannabis commercialization with self-harm or assault injuries, relative to state-months not adopting any medical or recreational cannabis legalization over the same period. Bars represent the corresponding 95% confidence intervals.

© 2021 Matthay EC et al. *JAMA Network Open*.

eFigure 4: Adjusted associations of medical and recreational cannabis commercialization with self-harm and assault injury rates, with recreational dispensary categories disaggregated by THC dosage-related restrictions, for the overall study population, 2003-2017

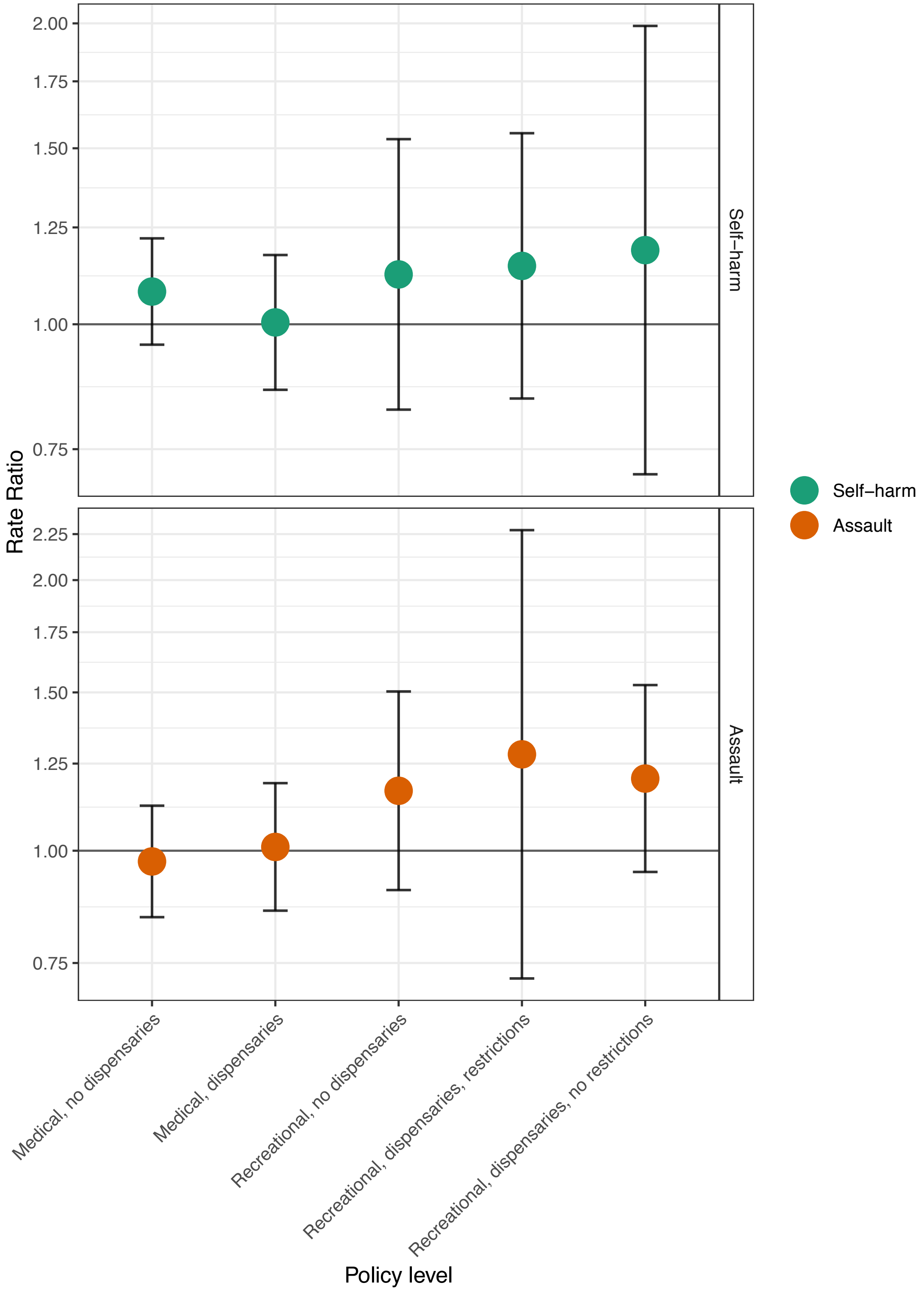

Legend: Points represent the estimated rate ratio for the association of each type of cannabis commercialization with self-harm or assault injuries, relative to state-months not adopting any medical or recreational cannabis legalization over the same period. Bars represent the corresponding 95% confidence intervals.

© 2021 Matthay EC et al. *JAMA Network Open*.

eFigure 5: Adjusted associations of medical and recreational cannabis commercialization with self-harm and assault injury rates, with medical and recreational policy categories collapsed, for the overall study population, 2003-2017

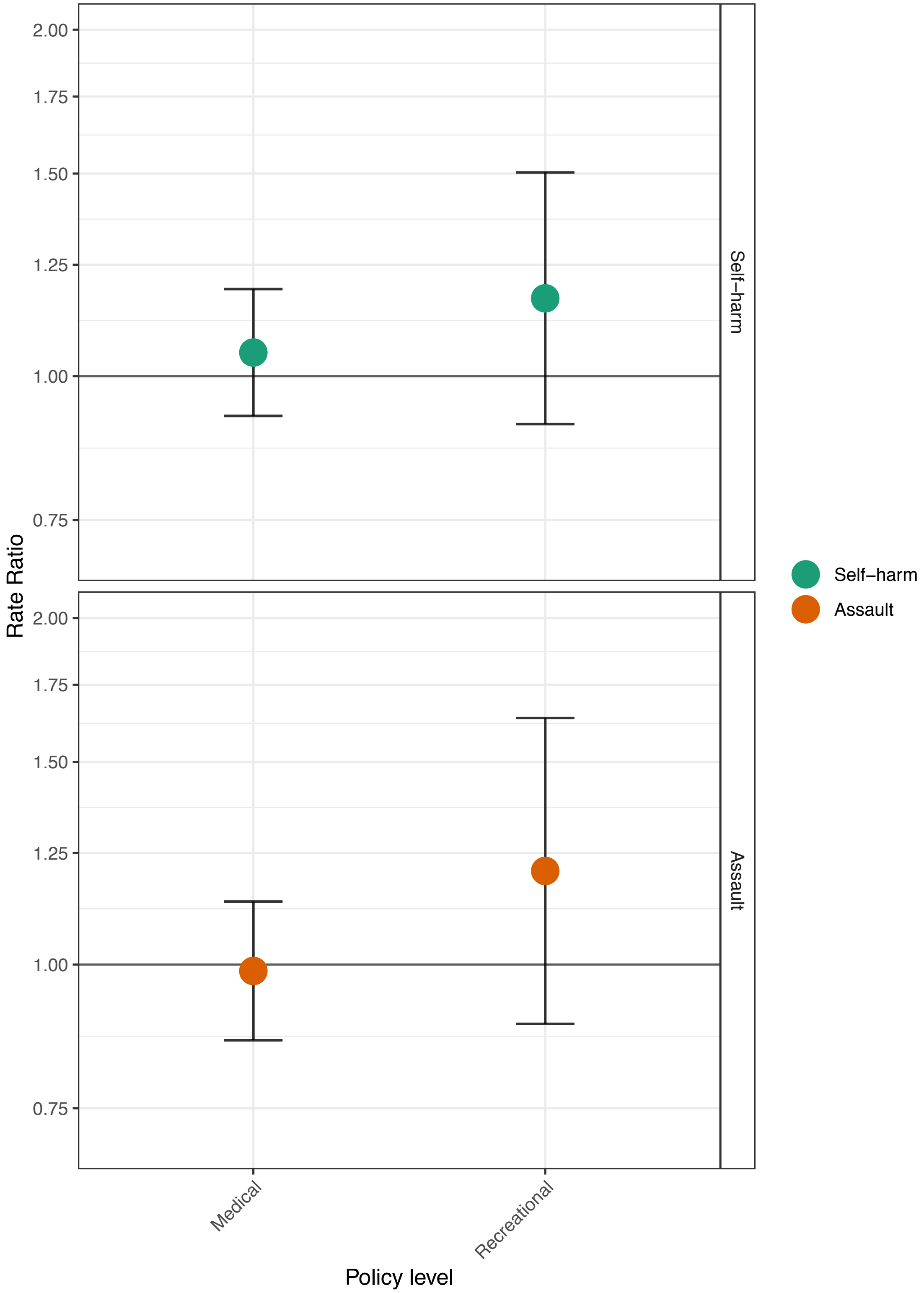

Legend: Points represent the estimated rate ratio for the association of each type of cannabis commercialization with self-harm or assault injuries, relative to state-months not adopting any medical or recreational cannabis legalization over the same period. Bars represent the corresponding 95% confidence intervals.

© 2021 Matthay EC et al. *JAMA Network Open*.

eFigure 6: Adjusted associations of medical and recreational cannabis commercialization with the all-claims rate as a negative control outcome, for the overall study population, 2003-2017

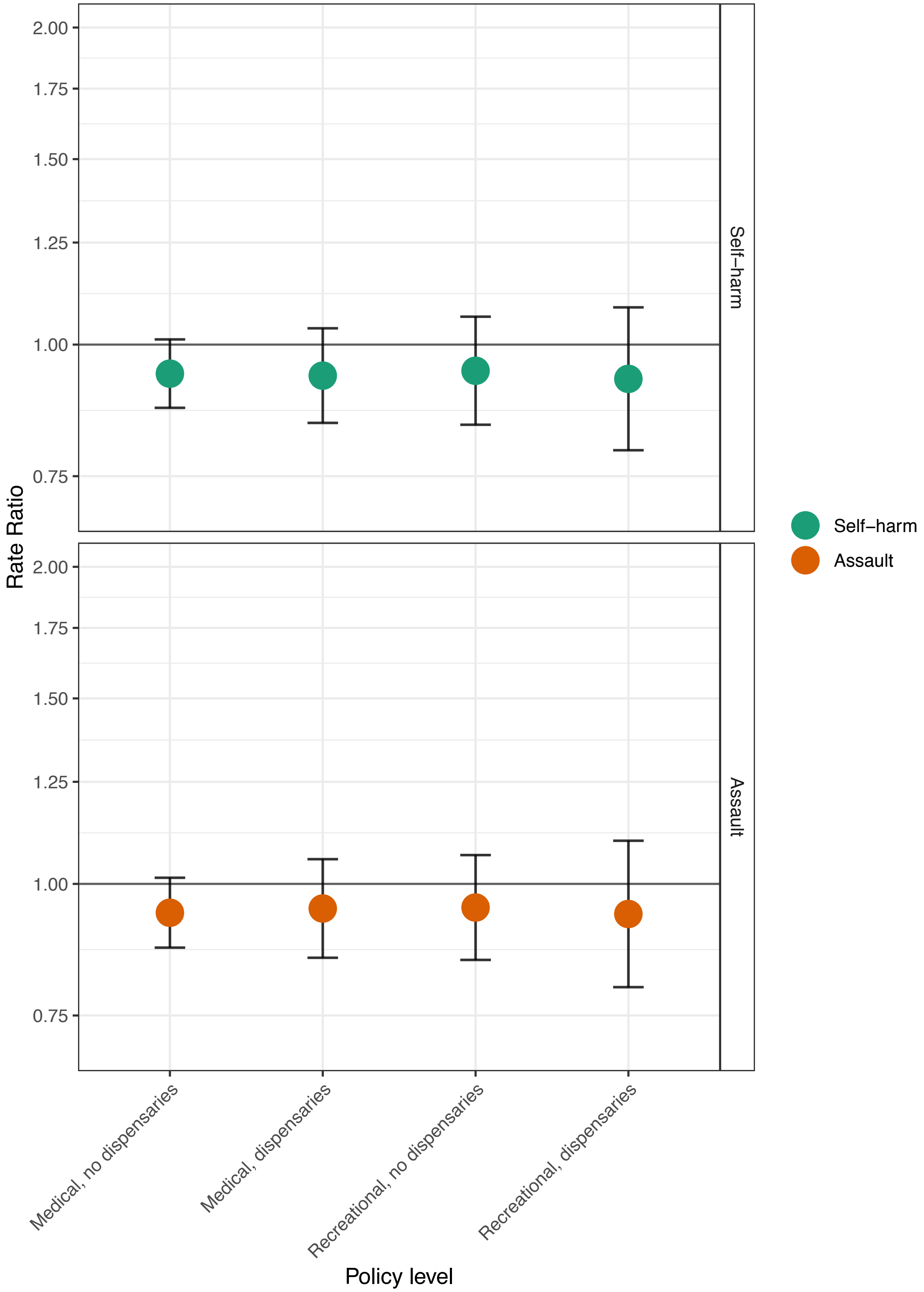

Legend: Points represent the estimated rate ratio for the association of each type of cannabis commercialization with self-harm or assault injuries, relative to state-months not adopting any medical or recreational cannabis legalization over the same period. Bars represent the corresponding 95% confidence intervals.

© 2021 Matthay EC et al. *JAMA Network Open*.

eFigure 7: Adjusted associations of naloxone overdose prevention laws as a negative control exposure with self-harm and assault injury rates, for the overall study population, 2003-2017

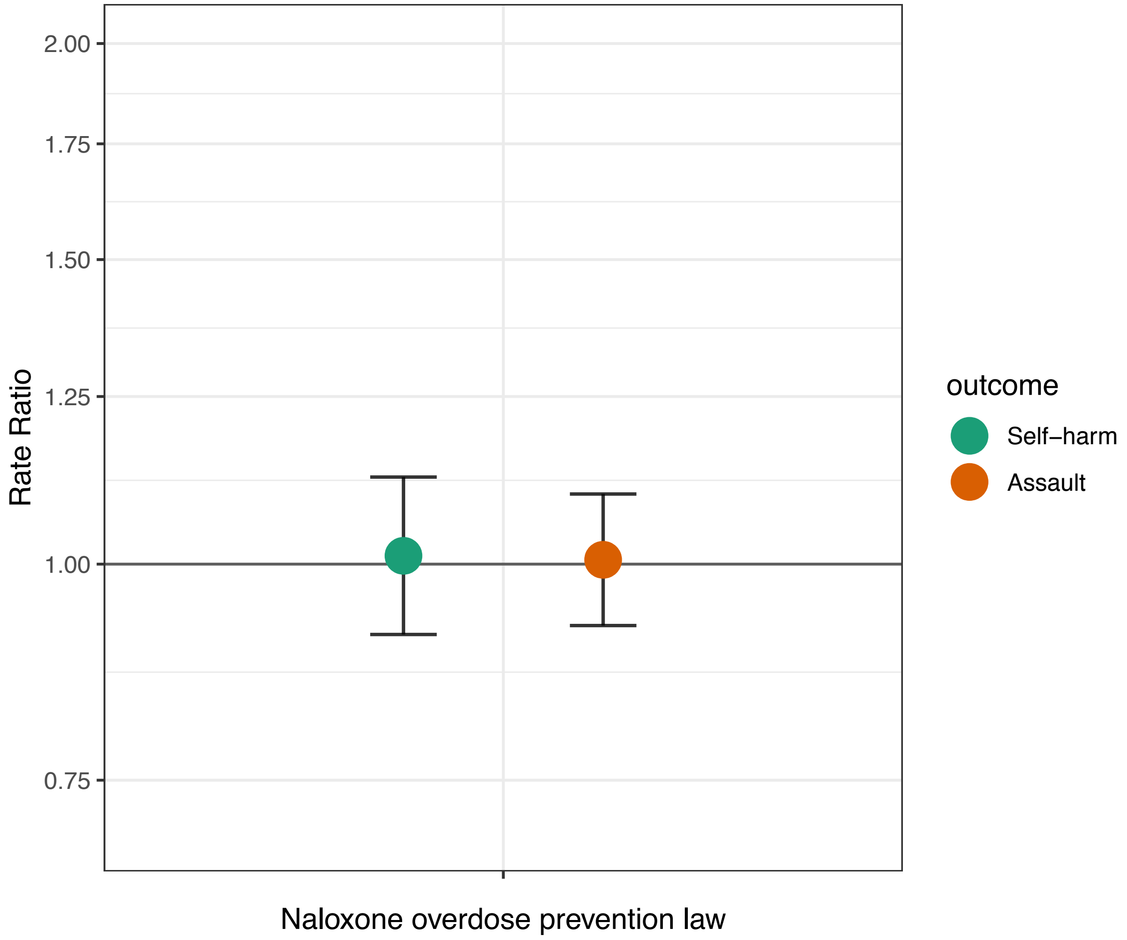

Legend: Points represent the estimated rate ratio for the association of naloxone overdose prevention laws (a negative control exposure) with self-harm or assault injuries, relative to state-months not adopting such laws over the same period. The bars represent the corresponding 95% confidence intervals.

© 2021 Matthay EC et al. *JAMA Network Open*.

eFigure 8: Adjusted associations of hypothetical, randomly generated law changes as a negative control exposure with self-harm and assault injury rates, for the overall study population, 2003-2017

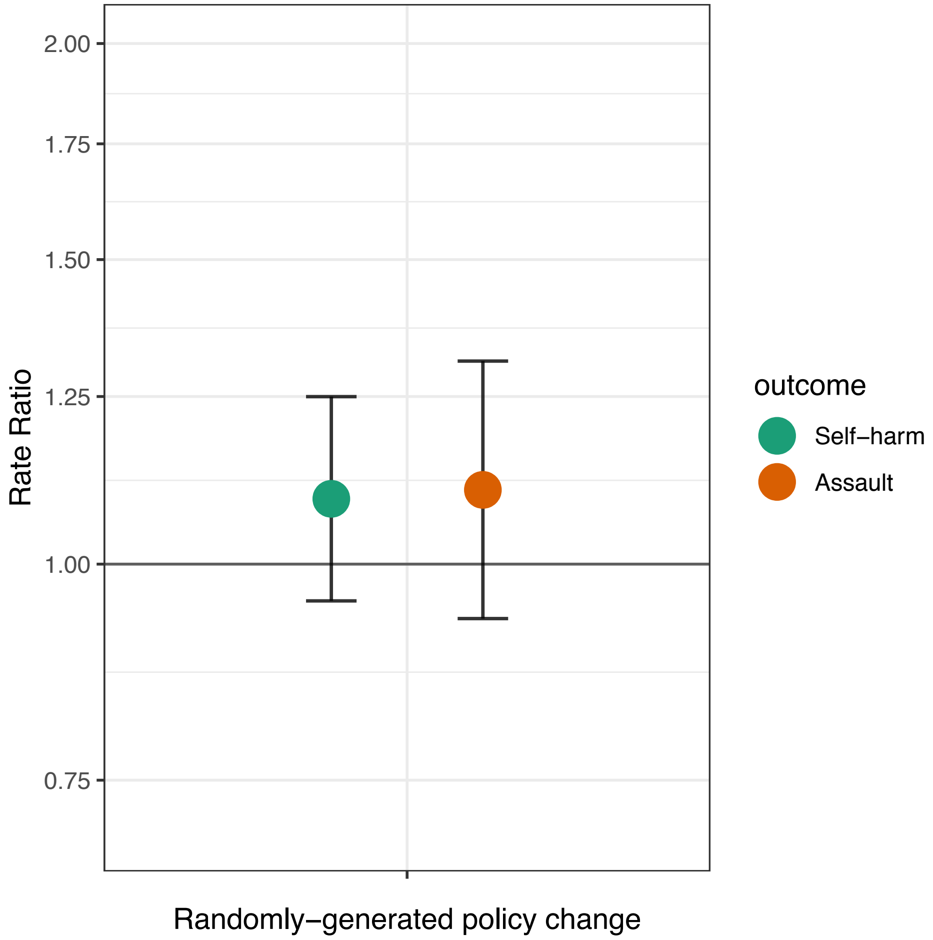

Legend: Points represent the estimated rate ratio for the association of a hypothetical, randomly generated policy change (a negative control exposure) with self-harm or assault injuries, relative to state-months not adopting such "laws" over the same period. The bars represent the corresponding 95% confidence intervals.

© 2021 Matthay EC et al. *JAMA Network Open*.

eFigure 9: Adjusted associations of medical and recreational cannabis commercialization with self-harm and assault injury rates, with additional adjustment for linear state-specific time trends, for the overall study population, 2003-2017

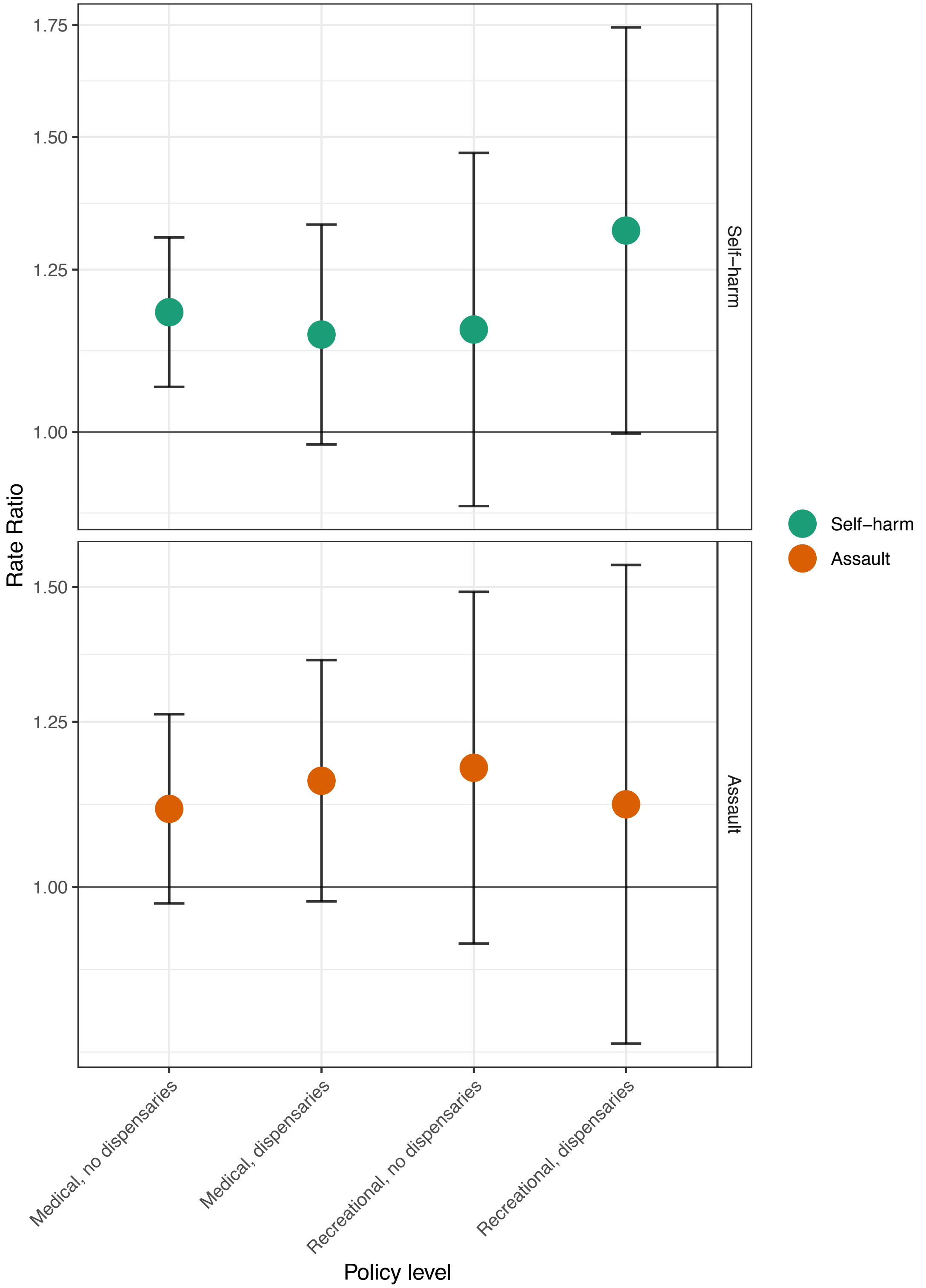

Legend: Points represent the estimated rate ratio for the association of each type of cannabis commercialization with self-harm or assault injuries, relative to state-months not adopting any medical or recreational cannabis legalization over the same period. Bars represent the corresponding 95% confidence intervals.

© 2021 Matthay EC et al. *JAMA Network Open*.

eFigure 10: Adjusted associations of medical and recreational cannabis commercialization with self-harm and assault injury rates, for states with cannabis legalization, for the overall study population, 2003-2017

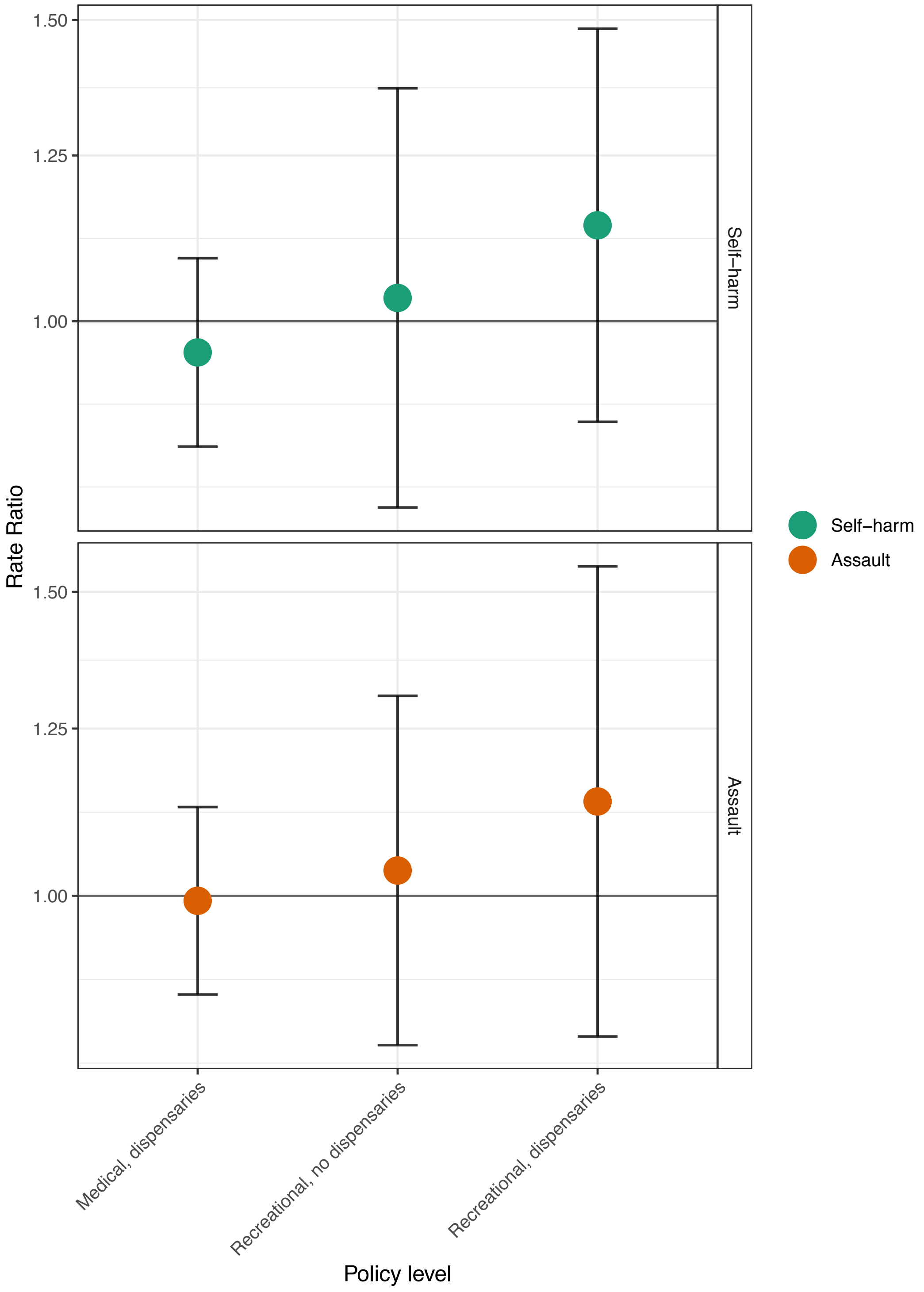

Legend: Points represent the estimated rate ratio for the association of each type of cannabis commercialization with self-harm or assault injuries, relative to state-months with medical cannabis without dispensaries. The primary analysis used all state-months 2003-2017, including state-months with and without cannabis legalization laws. This analysis used only state-months in which some form of cannabis legalization was effective, with the most stringent category (medical, without dispensaries) as the reference category. Bars represent the corresponding 95% confidence intervals.

© 2021 Matthay EC et al. *JAMA Network Open*.

eFigure 11: Adjusted associations of recreational (versus medical) cannabis legalization and commercialization (dispensaries versus home-grow only) with self-harm and assault injury rates, for states with cannabis legalization, for the overall study population, 2003-2017

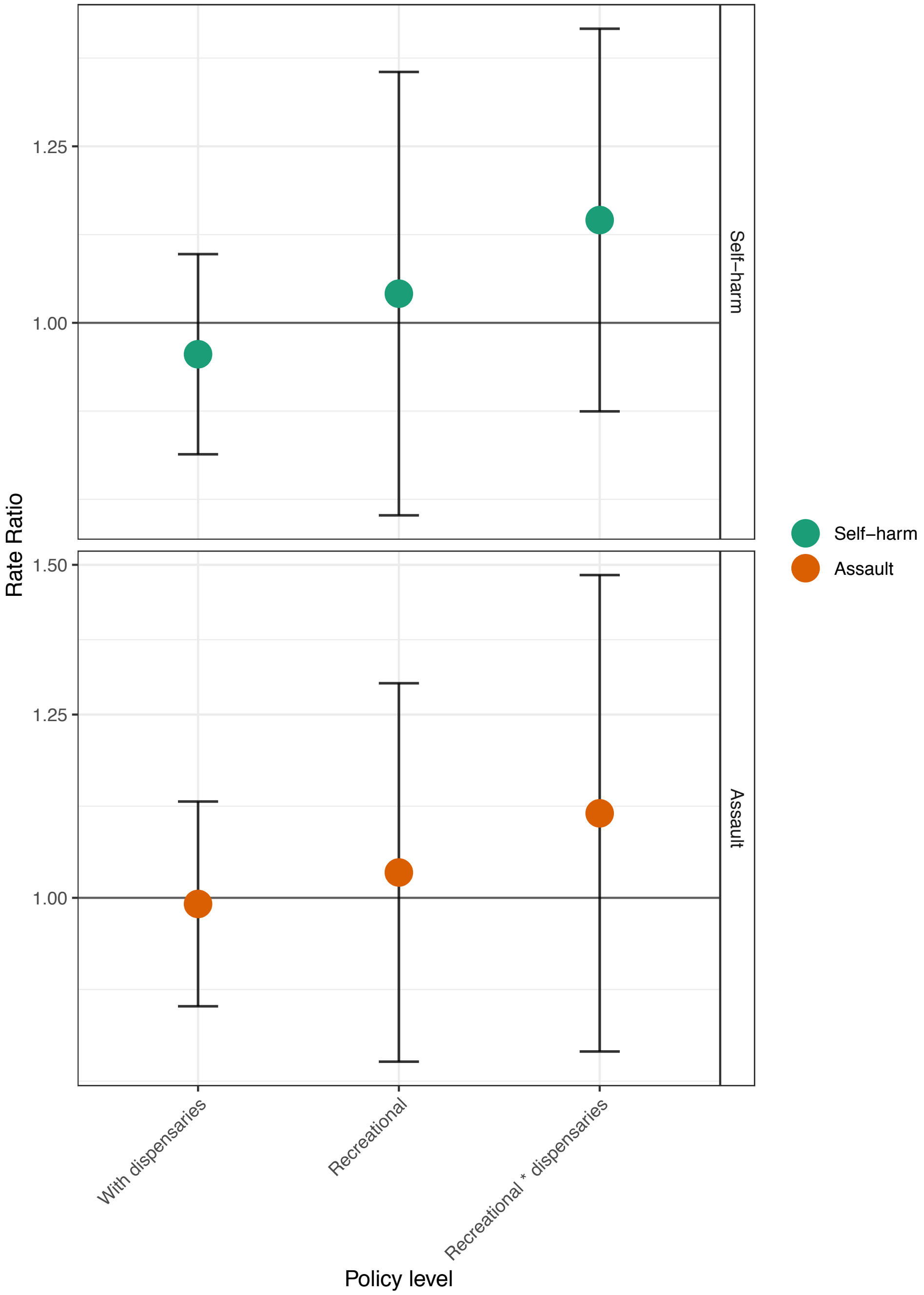

Legend: Points represent the estimated rate ratio for the association of each type of cannabis commercialization with self-harm or assault injuries, relative to state-months with medical cannabis without dispensaries. Bars represent the corresponding 95% confidence intervals. The primary analysis used all state-months 2003-2017, including state-months with and without cannabis legalization laws. This analysis used only state-months in which some form of cannabis legalization was effective, with the most stringent category (medical, without dispensaries) as the reference category. Commercialization refers to the presence of dispensaries in either medical or recreational cannabis states. “Recreational with dispensaries” refers to the estimate for an interaction term between recreation (versus medical) legalization and commercialization. This analytic specification was not tested for analyses by age group and gender because such analyses effectively involve third-order interactions and were under powered.

© 2021 Matthay EC et al. *JAMA Network Open*.

eFigure 12: Adjusted associations of medical and recreational cannabis commercialization with assault injury rates, using unique claims as the denominator for outcome rates, by age group and gender, 2003-2017

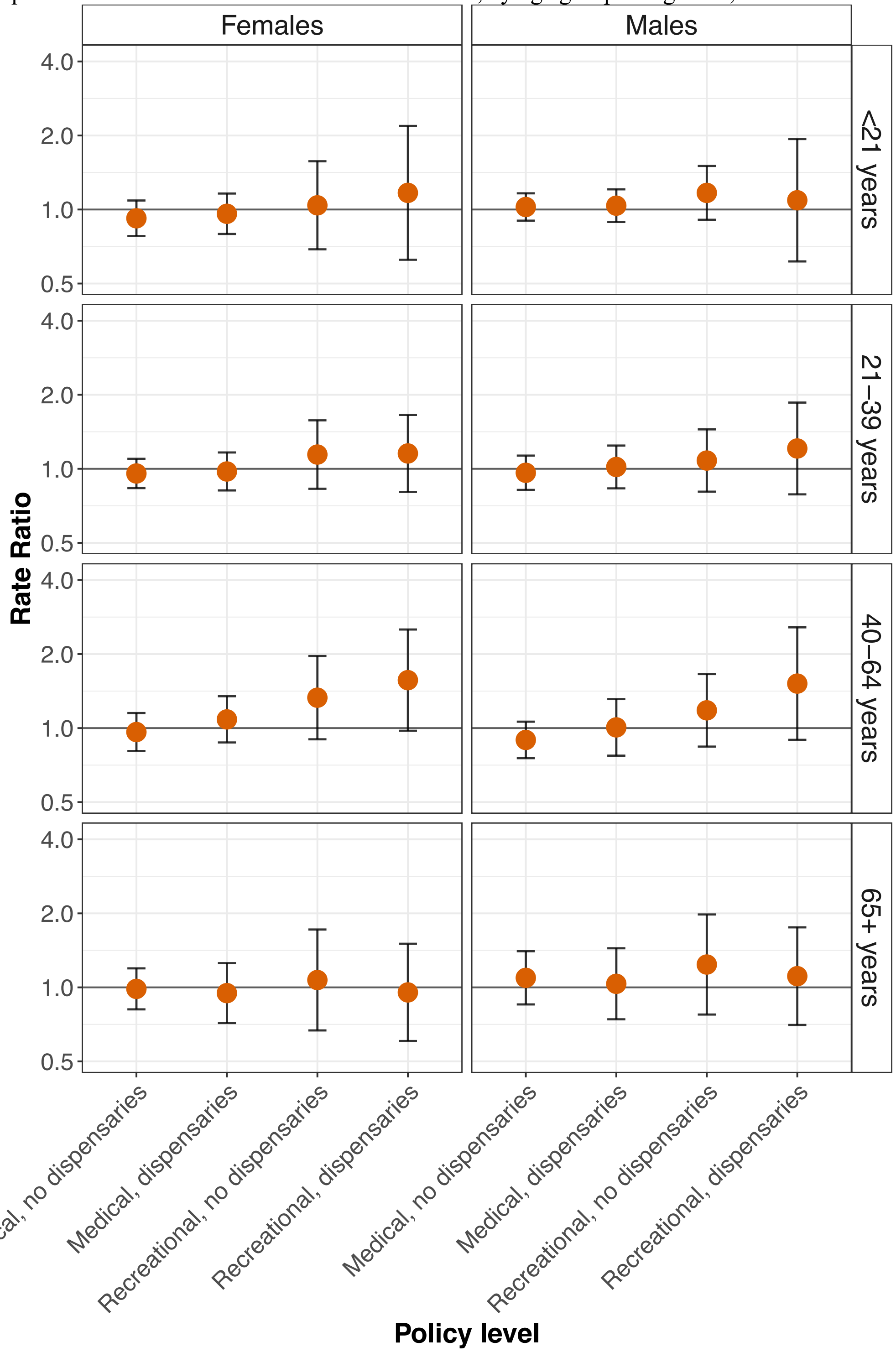

Legend: Points represent the estimated rate ratio for the association of each type of cannabis commercialization with assault injuries, relative to state-months not adopting any medical or recreational cannabis legalization over the same period. Bars represent the corresponding 95% confidence intervals. The primary analysis used the number of insured beneficiaries in the given state and month as the denominator when calculating rates of self-harm and assault. In this sensitivity analysis, we used unique claims (the number of unique claims made per state-month) as the denominator, to account for changes in overall utilization over time.

© 2021 Matthay EC et al. *JAMA Network Open*.

eFigure 13: Adjusted associations of medical and recreational cannabis commercialization with self-harm injury rates, using unique claims as the denominator for outcome rates, by age group and gender, 2003-2017

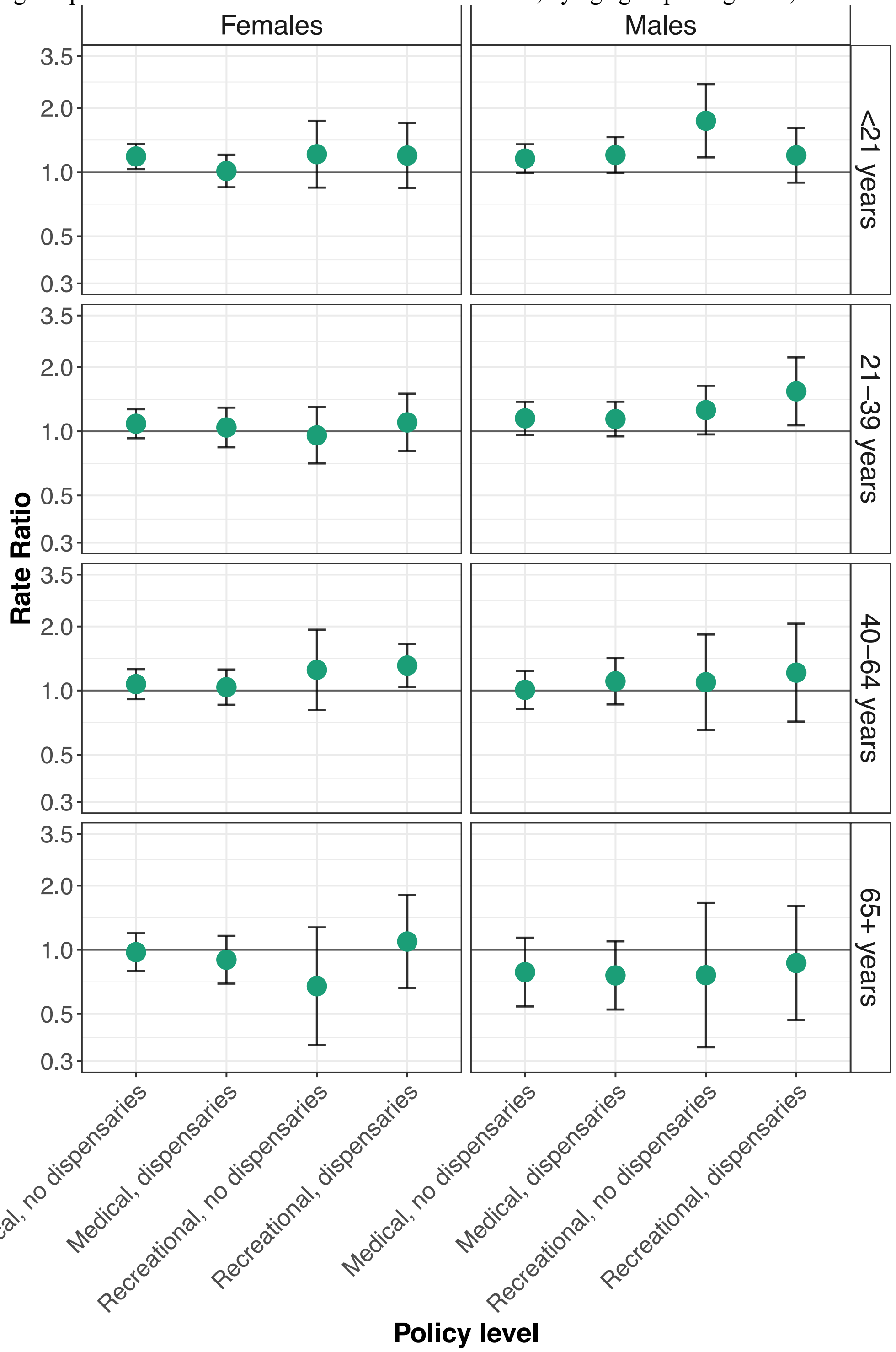

Legend: Points represent the estimated rate ratio for the association of each type of cannabis commercialization with self-harm injuries, relative to state-months not adopting any medical or recreational cannabis legalization over the same period. Bars represent the corresponding 95% confidence intervals. The primary analysis used the number of insured beneficiaries in the given state and month as the denominator when calculating rates of self-harm and assault. In this sensitivity analysis, we used unique claims (the number of unique claims made per state-month) as the denominator, to account for changes in overall utilization over time.

© 2021 Matthay EC et al. *JAMA Network Open*.

eFigure 14: Adjusted associations of medical and recreational cannabis commercialization with assault injury rates, using active members as the denominator for outcome rates, by age group and gender, 2003-2017

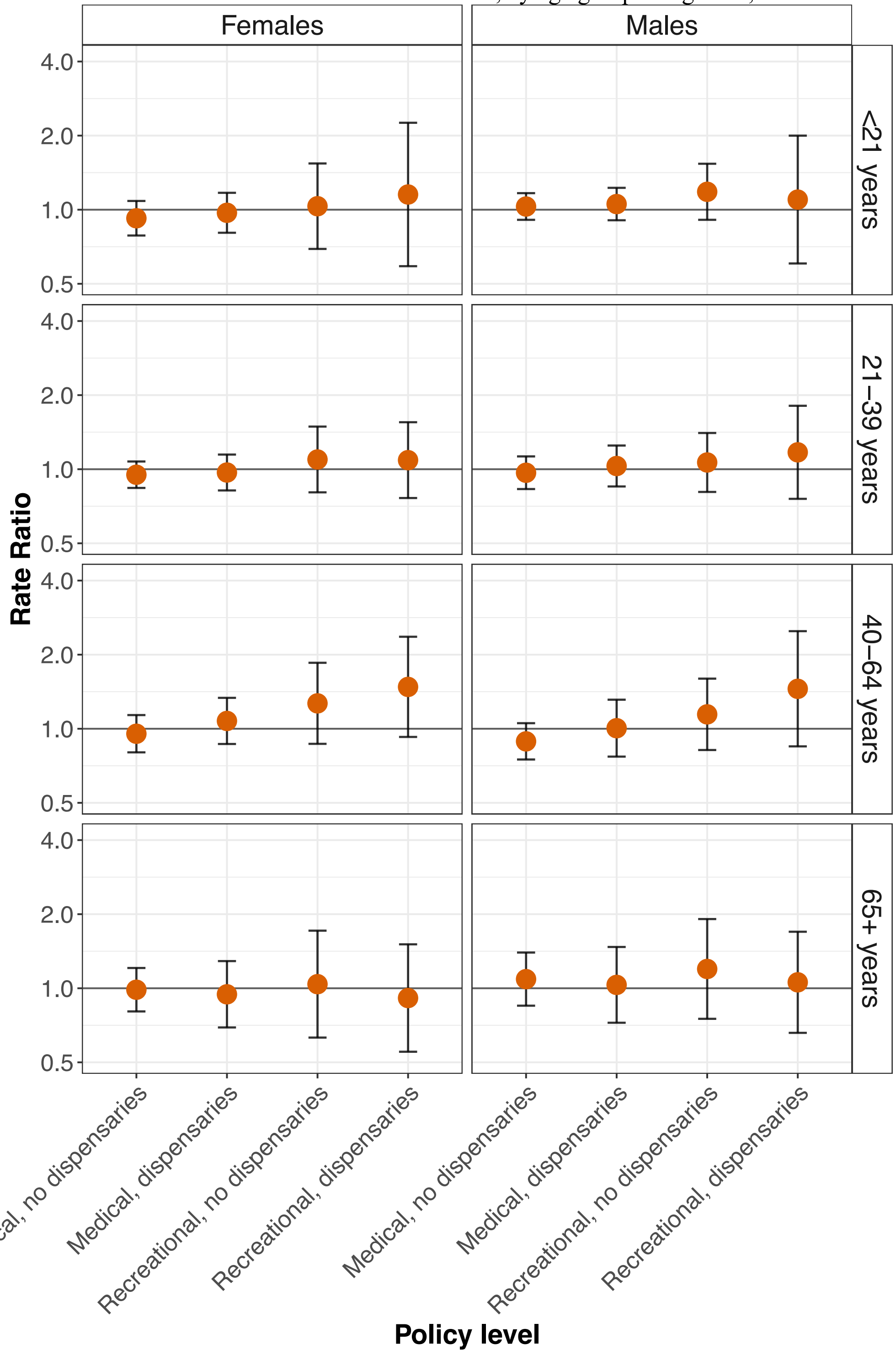

Legend: Points represent the estimated rate ratio for the association of each type of cannabis commercialization with assault injuries, relative to state-months not adopting any medical or recreational cannabis legalization over the same period. Bars represent the corresponding 95% confidence intervals. The primary analysis used the number of insured beneficiaries in the given state and month as the denominator when calculating rates of self-harm and assault. In this sensitivity analysis, we used active members (the number of unique members who filed at least one claim per state month), because this approach is more robust to high-utilizing members.

© 2021 Matthay EC et al. *JAMA Network Open*.

eFigure 15: Adjusted associations of medical and recreational cannabis commercialization with self-harm injury rates, using active members as the denominator for outcome rates, by age group and gender, 2003-2017

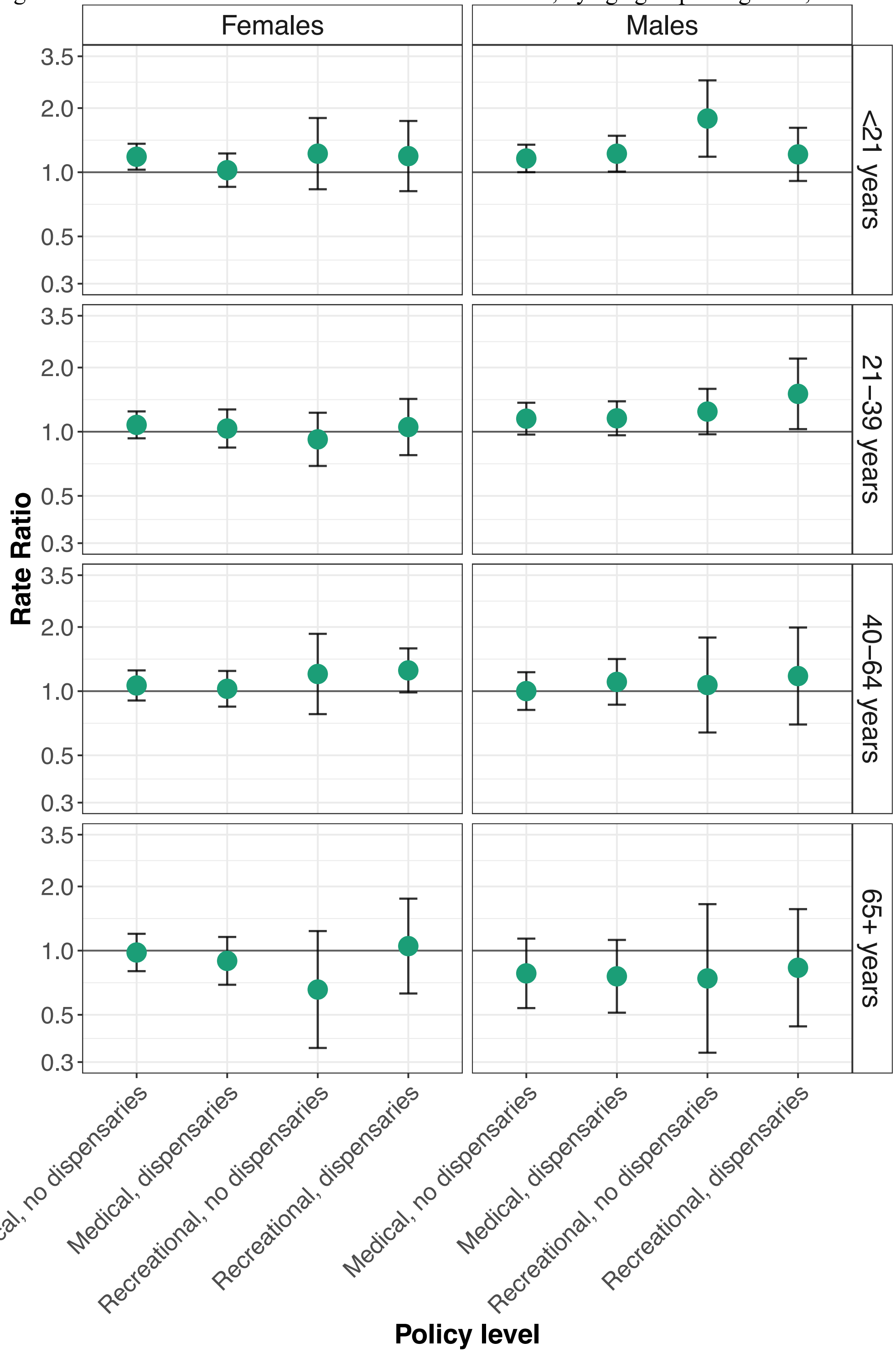

Legend: Points represent the estimated rate ratio for the association of each type of cannabis commercialization with self-harm injuries, relative to state-months not adopting any medical or recreational cannabis legalization over the same period. Bars represent the corresponding 95% confidence intervals. The primary analysis used the number of insured beneficiaries in the given state and month as the denominator when calculating rates of self-harm and assault. In this sensitivity analysis, we used active members (the number of unique members who filed at least one claim per state month), because this approach is more robust to high-utilizing members.

© 2021 Matthay EC et al. *JAMA Network Open*.

eFigure 16: Adjusted associations of medical and recreational cannabis commercialization with assault injury rates, adjusted for firearm availability, without the District of Columbia, by age group and gender, 2003-2017

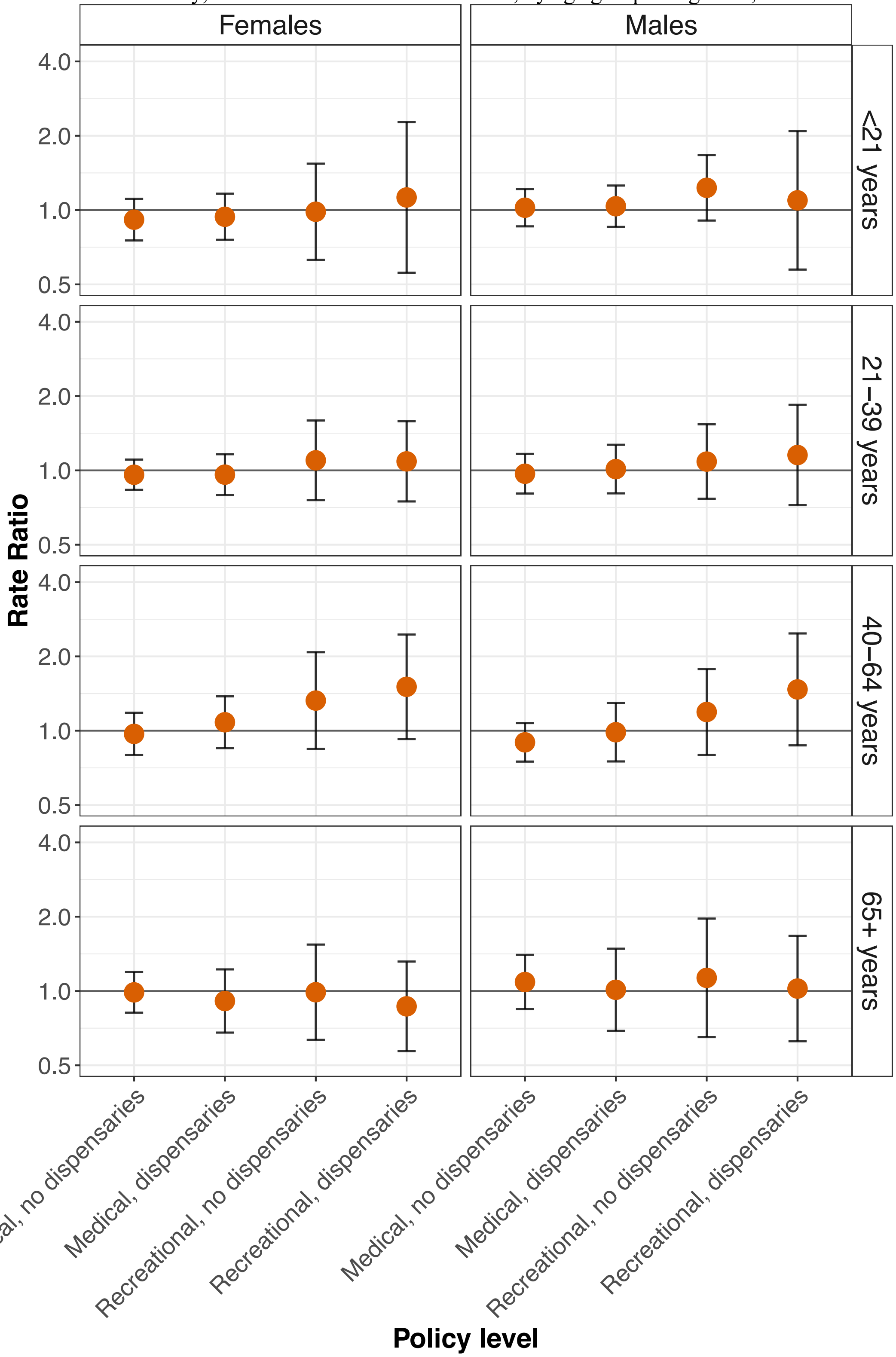

Legend: Points represent the estimated rate ratio for the association of each type of cannabis commercialization with assault injuries, relative to state-months not adopting any medical or recreational cannabis legalization over the same period. Bars represent the corresponding 95% confidence intervals.

© 2021 Matthay EC et al. *JAMA Network Open*.

eFigure 17: Adjusted associations of medical and recreational cannabis commercialization with self-harm injury rates, adjusted for firearm availability, without the District of Columbia, by age group and gender, 2003-2017

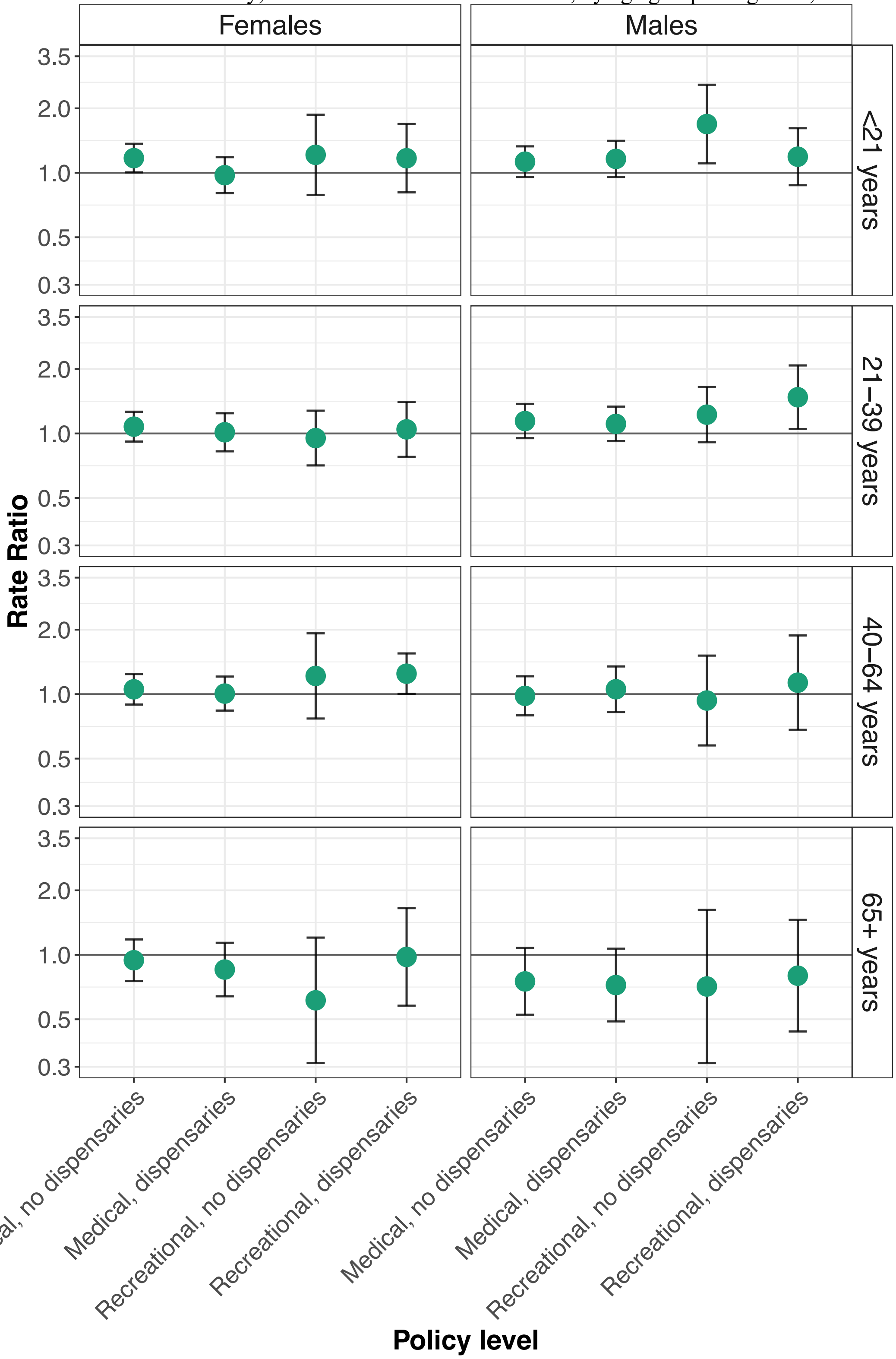

Legend: Points represent the estimated rate ratio for the association of each type of cannabis commercialization with self-harm injuries, relative to state-months not adopting any medical or recreational cannabis legalization over the same period. Bars represent the corresponding 95% confidence intervals.

© 2021 Matthay EC et al. *JAMA Network Open*.

eFigure 18: Adjusted associations of medical and recreational cannabis commercialization with assault injury rates, with recreational dispensary categories disaggregated by THC dosage-related restrictions, by age group and gender, 2003-2017

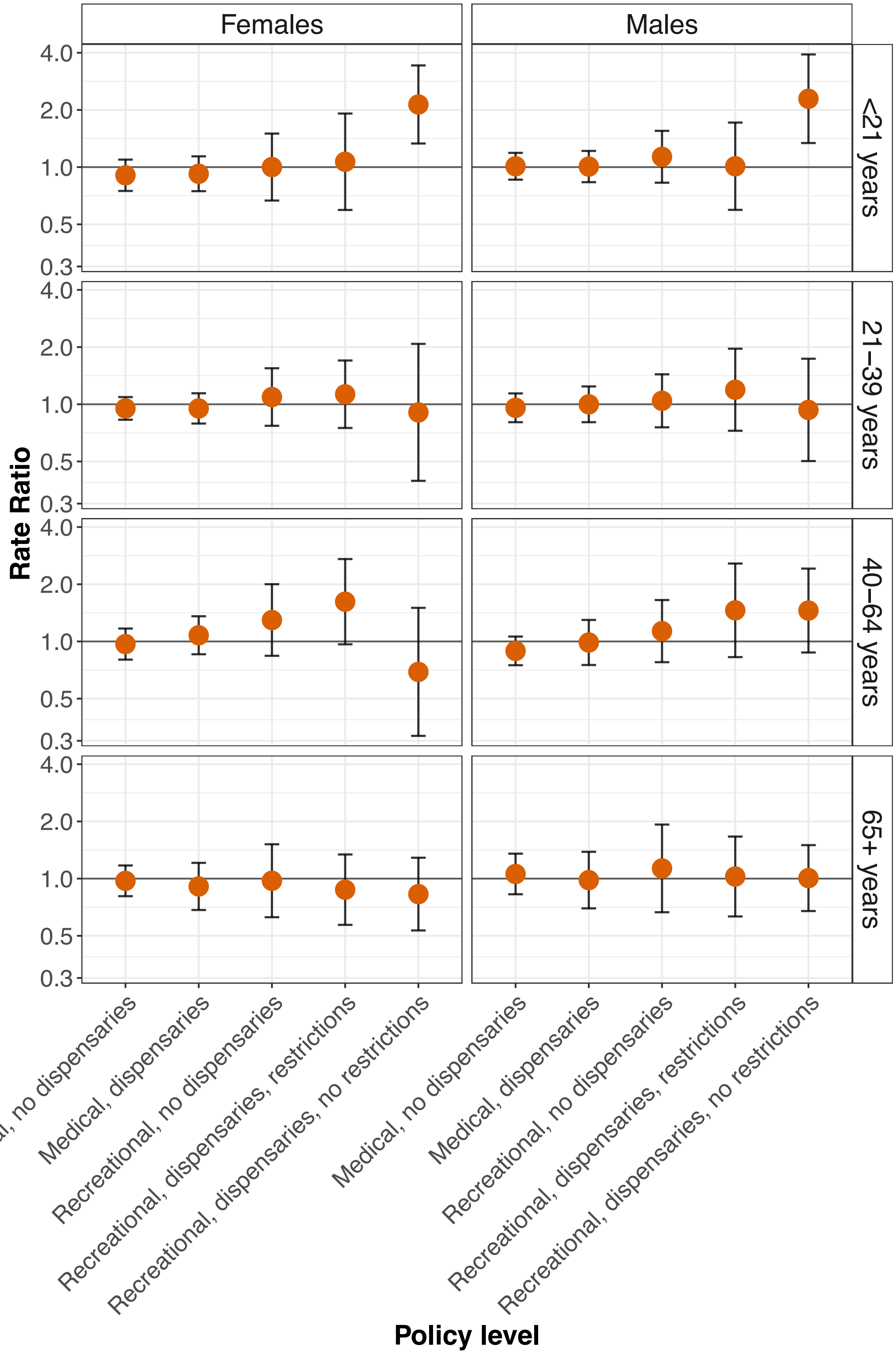

Legend: Points represent the estimated rate ratio for the association of each type of cannabis commercialization with assault injuries, relative to state-months not adopting any medical or recreational cannabis legalization over the same period. Bars represent the corresponding 95% confidence intervals.

© 2021 Matthay EC et al. *JAMA Network Open*.

eFigure 19: Adjusted associations of medical and recreational cannabis commercialization with self-harm injury rates, with recreational dispensary categories disaggregated by THC dosage-related restrictions, by age group and gender, 2003-2017

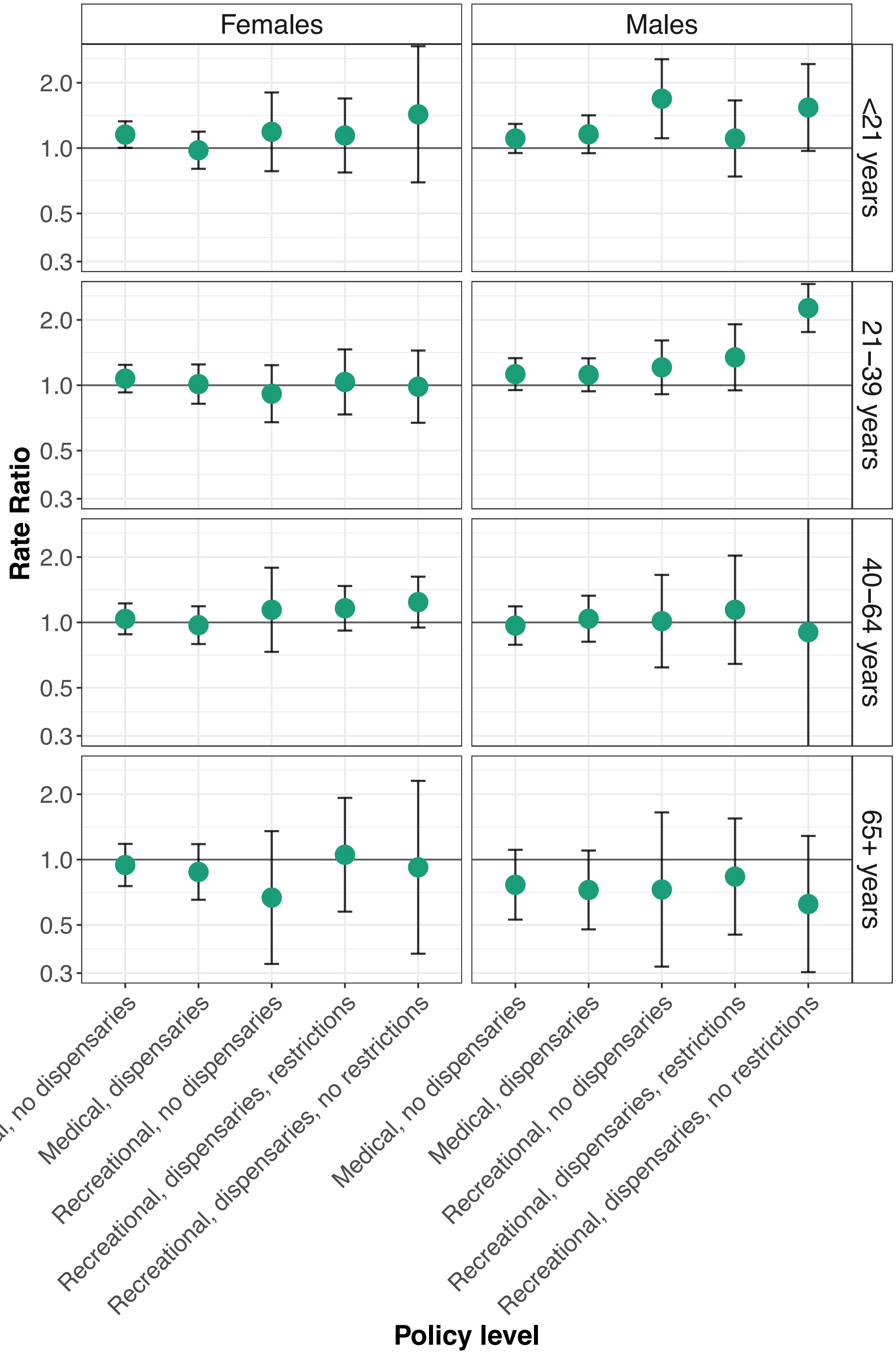

Legend: Points represent the estimated rate ratio for the association of each type of cannabis commercialization with self-harm injuries, relative to state-months not adopting any medical or recreational cannabis legalization over the same period. Bars represent the corresponding 95% confidence intervals.

© 2021 Matthay EC et al. *JAMA Network Open*.

eFigure 20: Adjusted associations of medical and recreational cannabis commercialization with assault injury rates, with medical and recreational policy categories collapsed, by age group and gender, 2003-2017

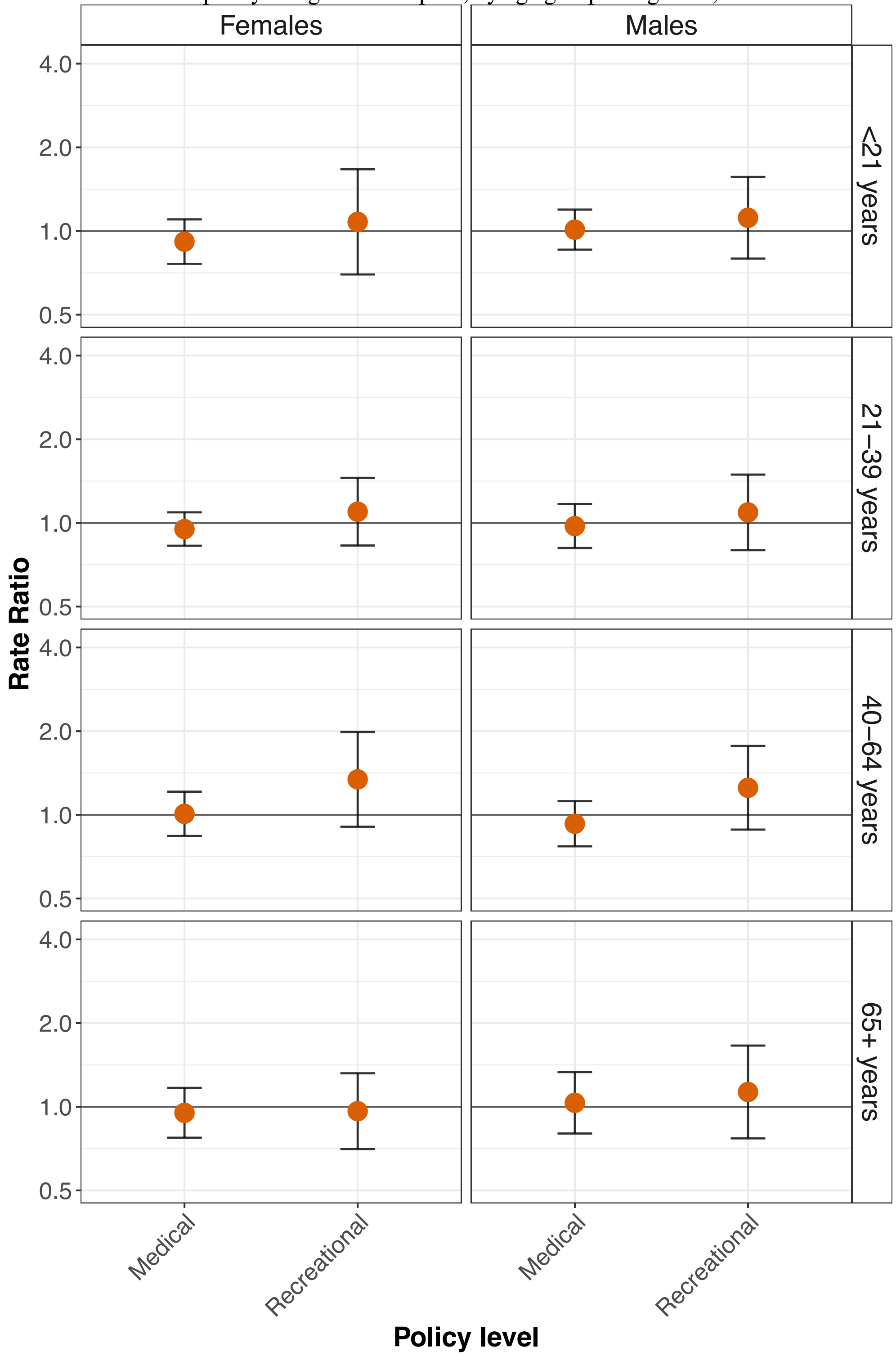

Legend: Points represent the estimated rate ratio for the association of each type of cannabis commercialization with assault injuries, relative to state-months not adopting any medical or recreational cannabis legalization over the same period. Bars represent the corresponding 95% confidence intervals.

© 2021 Matthay EC et al. *JAMA Network Open*.

eFigure 21: Adjusted associations of medical and recreational cannabis commercialization with self-harm injury rates, with medical and recreational policy categories collapsed, by age group and gender, 2003-2017

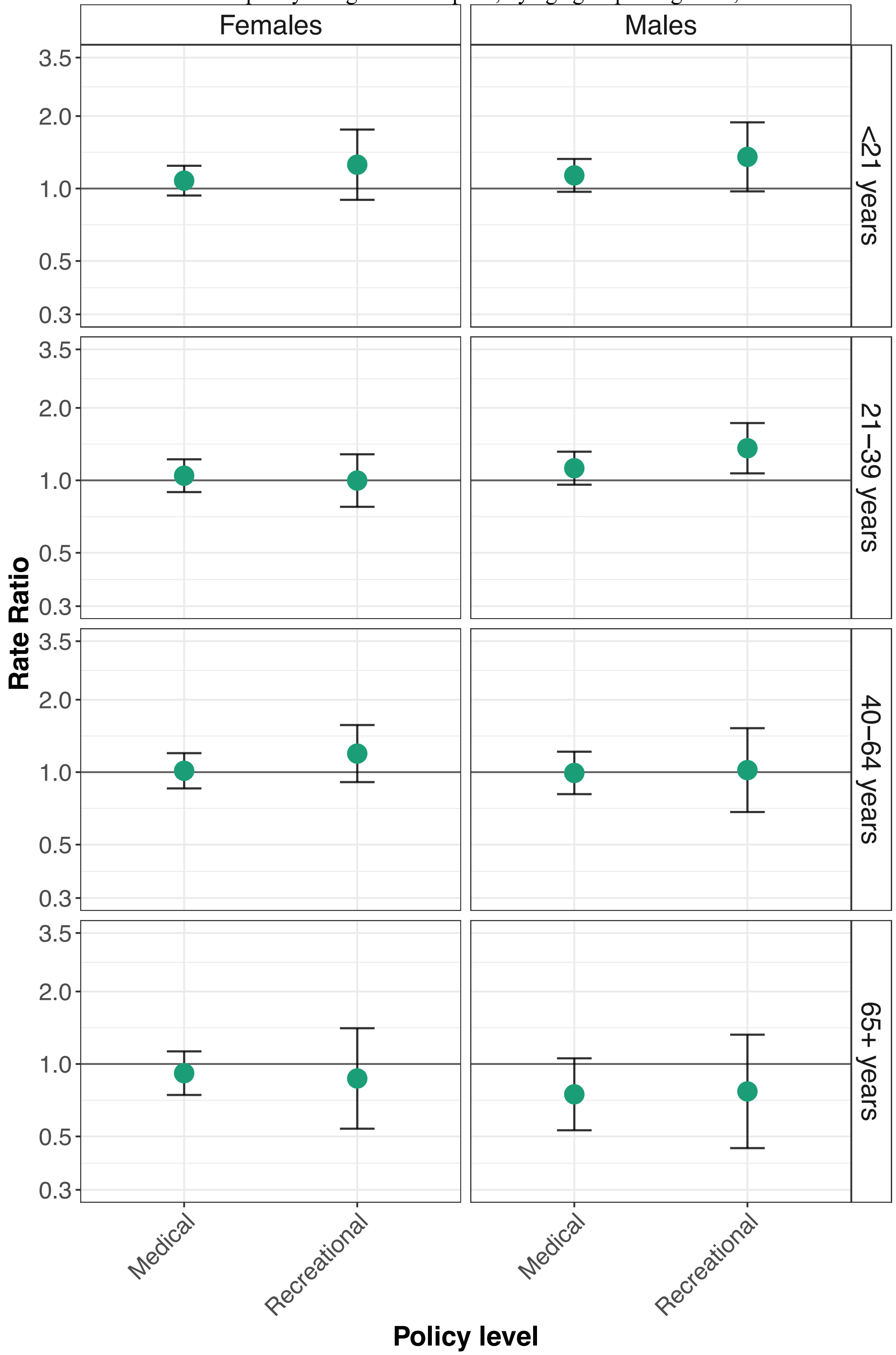

Legend: Points represent the estimated rate ratio for the association of each type of cannabis commercialization with self-harm injuries, relative to state-months not adopting any medical or recreational cannabis legalization over the same period. Bars represent the corresponding 95% confidence intervals.

© 2021 Matthay EC et al. *JAMA Network Open*.

eFigure 22: Adjusted associations of naloxone overdose prevention laws as a negative control exposure with assault injury rates, by age group and gender, 2003-2017

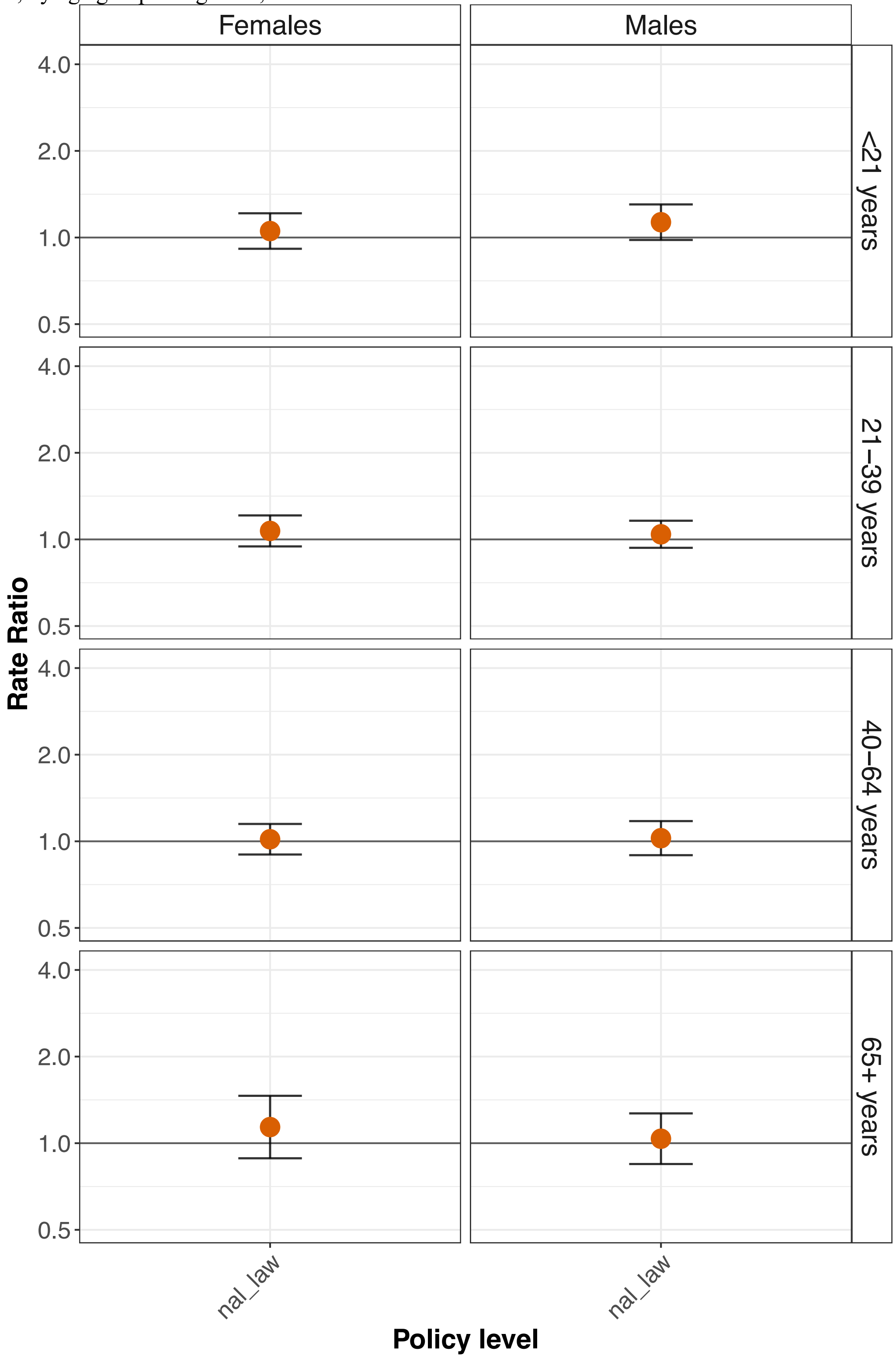

Legend: Points represent the estimated rate ratio for the association of naloxone overdose prevention laws (a negative control exposure) with assault injuries, relative to state-months not adopting such laws over the same period. The bars represent the corresponding 95% confidence intervals.

© 2021 Matthay EC et al. *JAMA Network Open*.

eFigure 23: Adjusted associations of naloxone overdose prevention laws as a negative control exposure with self-harm injury rates, by age group and gender, 2003-2017

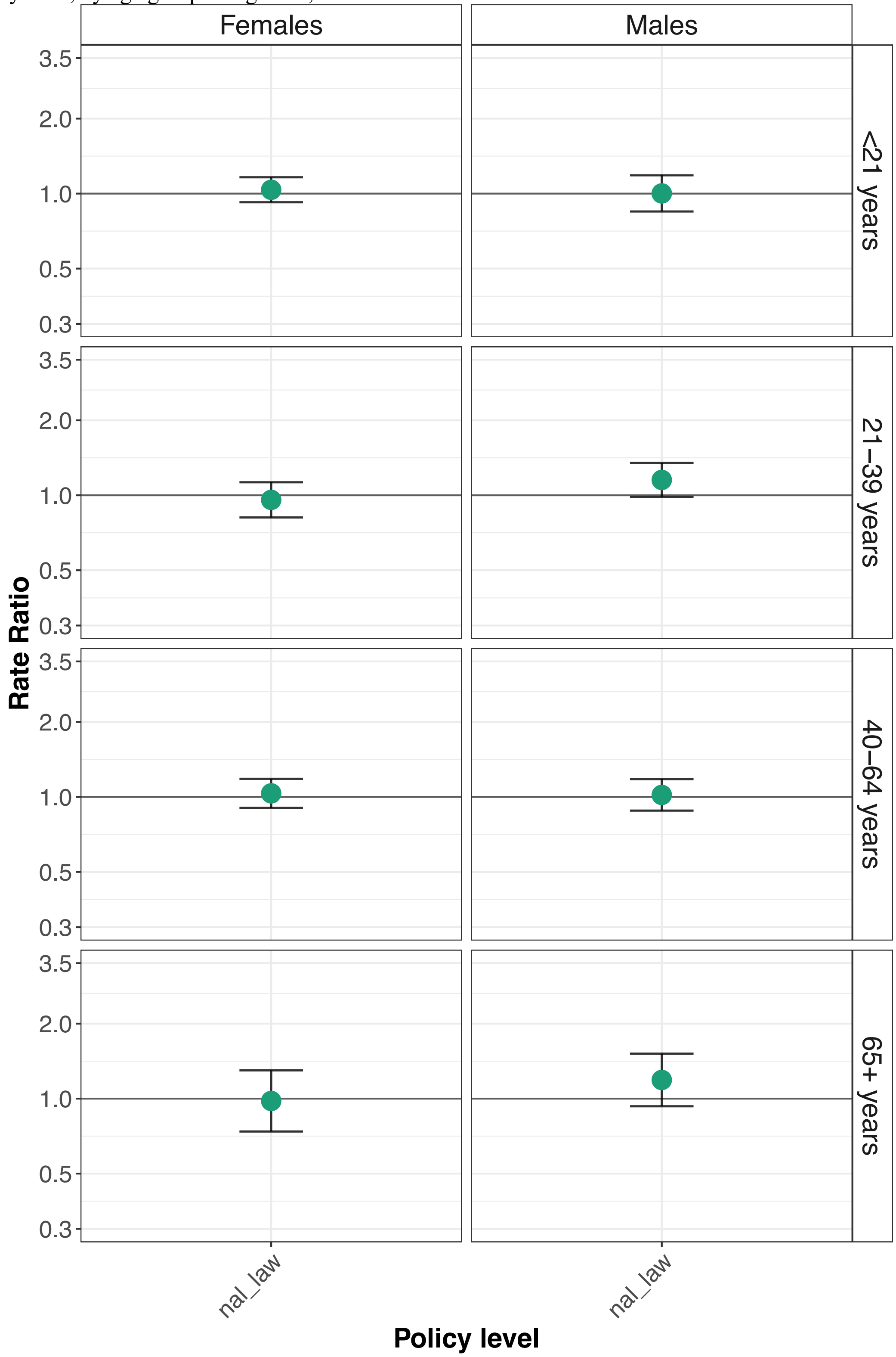

Legend: Points represent the estimated rate ratio for the association of naloxone overdose prevention laws (a negative control exposure) with self-harm injuries, relative to state-months not adopting such laws over the same period. The bars represent the corresponding 95% confidence intervals.

© 2021 Matthay EC et al. *JAMA Network Open*.

eFigure 24: Adjusted associations of hypothetical, randomly generated law changes as a negative control exposure with assault injury rates, by age group and gender, 2003-2017

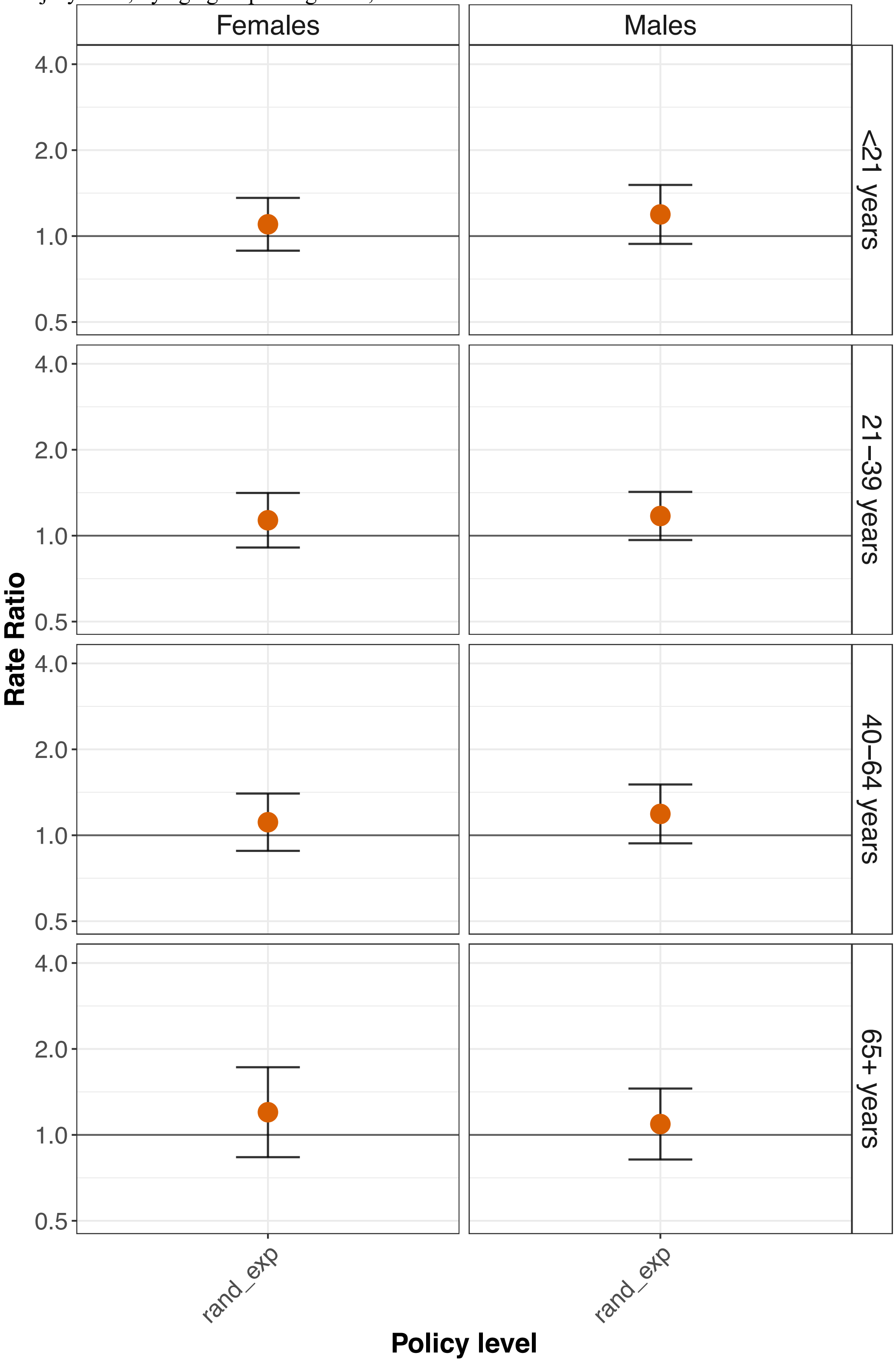

Legend: Points represent the estimated rate ratio for the association of a hypothetical, randomly generated policy change (a negative control exposure) with assault injuries, relative to state-months not adopting such "laws" over the same period. The bars represent the corresponding 95% confidence intervals.

© 2021 Matthay EC et al. *JAMA Network Open*.

eFigure 25: Adjusted associations of hypothetical, randomly generated law changes as a negative control exposure with self-harm injury rates, by age group and gender, 2003-2017

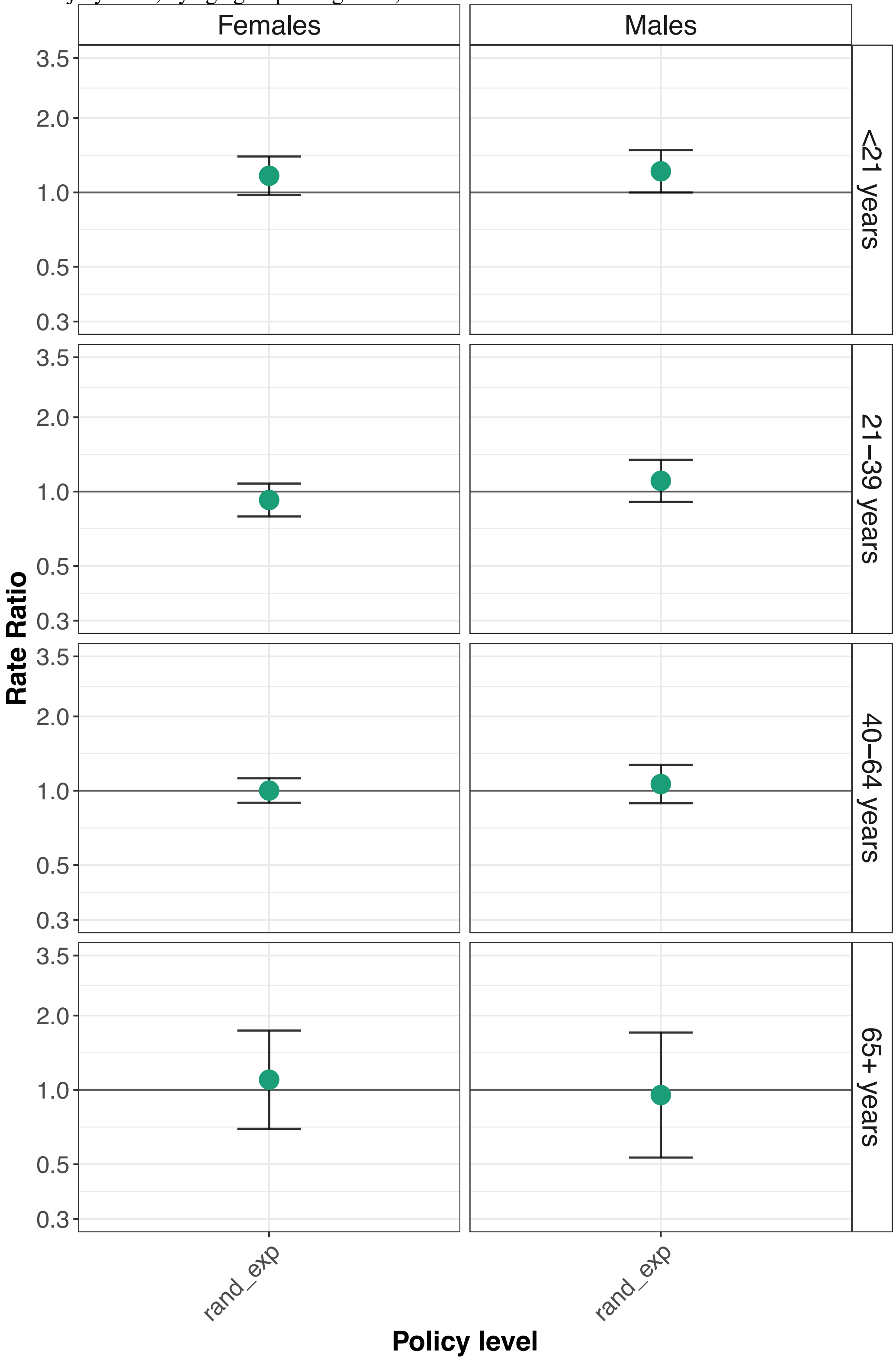

Legend: Points represent the estimated rate ratio for the association of a hypothetical, randomly generated policy change (a negative control exposure) with self-harm injuries, relative to state-months not adopting such "laws" over the same period. The bars represent the corresponding 95% confidence intervals.

© 2021 Matthay EC et al. *JAMA Network Open*.

eFigure 26: Adjusted associations of medical and recreational cannabis commercialization with assault injury rates, with additional adjustment for linear state-specific time trends, by age group and gender, 2003-2017

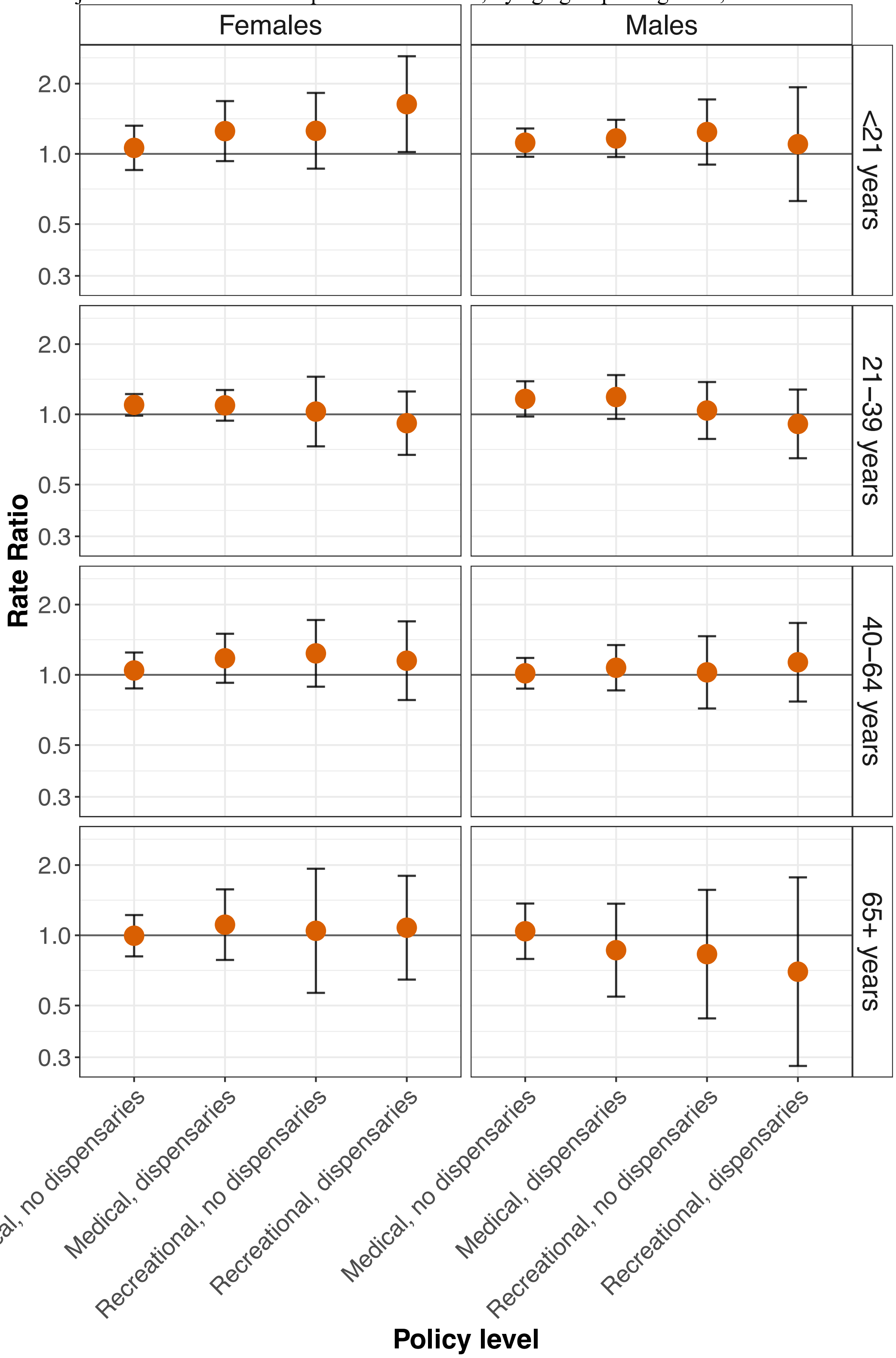

Legend: Points represent the estimated rate ratio for the association of each type of cannabis commercialization with assault injuries, relative to state-months not adopting any medical or recreational cannabis legalization over the same period. Bars represent the corresponding 95% confidence intervals.

© 2021 Matthay EC et al. *JAMA Network Open*.

eFigure 27: Adjusted associations of medical and recreational cannabis commercialization with self-harm injury rates, with additional adjustment for linear state-specific time trends, by age group and gender, 2003-2017

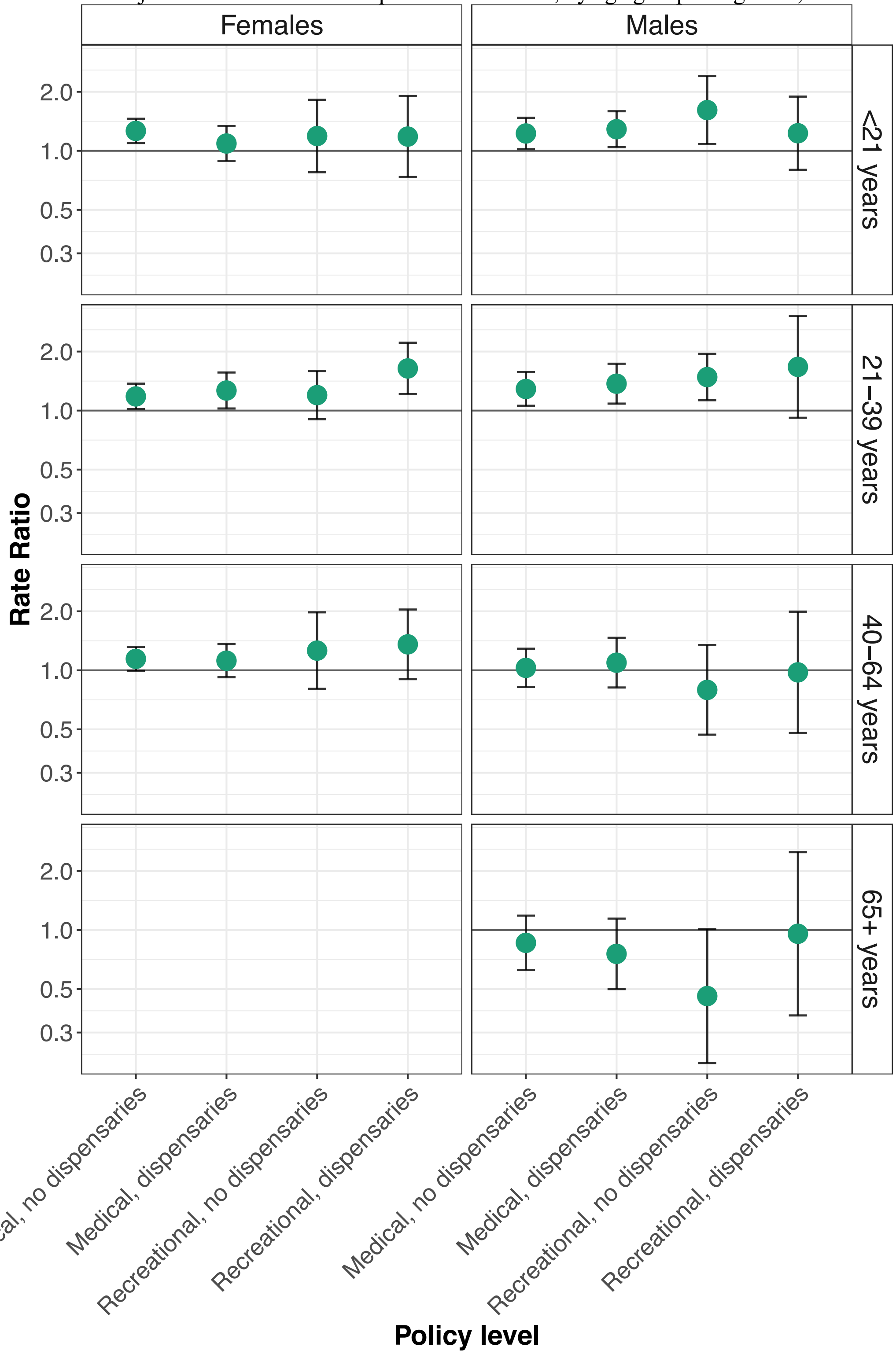

Legend: Points represent the estimated rate ratio for the association of each type of cannabis commercialization with self-harm injuries, relative to state-months not adopting any medical or recreational cannabis legalization over the same period. Bars represent the corresponding 95% confidence intervals. Self-harm claims for women aged 65 and older were very rare; results for this age-gender group were unstable and are not reported.

© 2021 Matthay EC et al. *JAMA Network Open*.

eFigure 28: Adjusted associations of medical and recreational cannabis commercialization with assault injury rates, for states with cannabis legalization, by age group and gender, 2003-2017

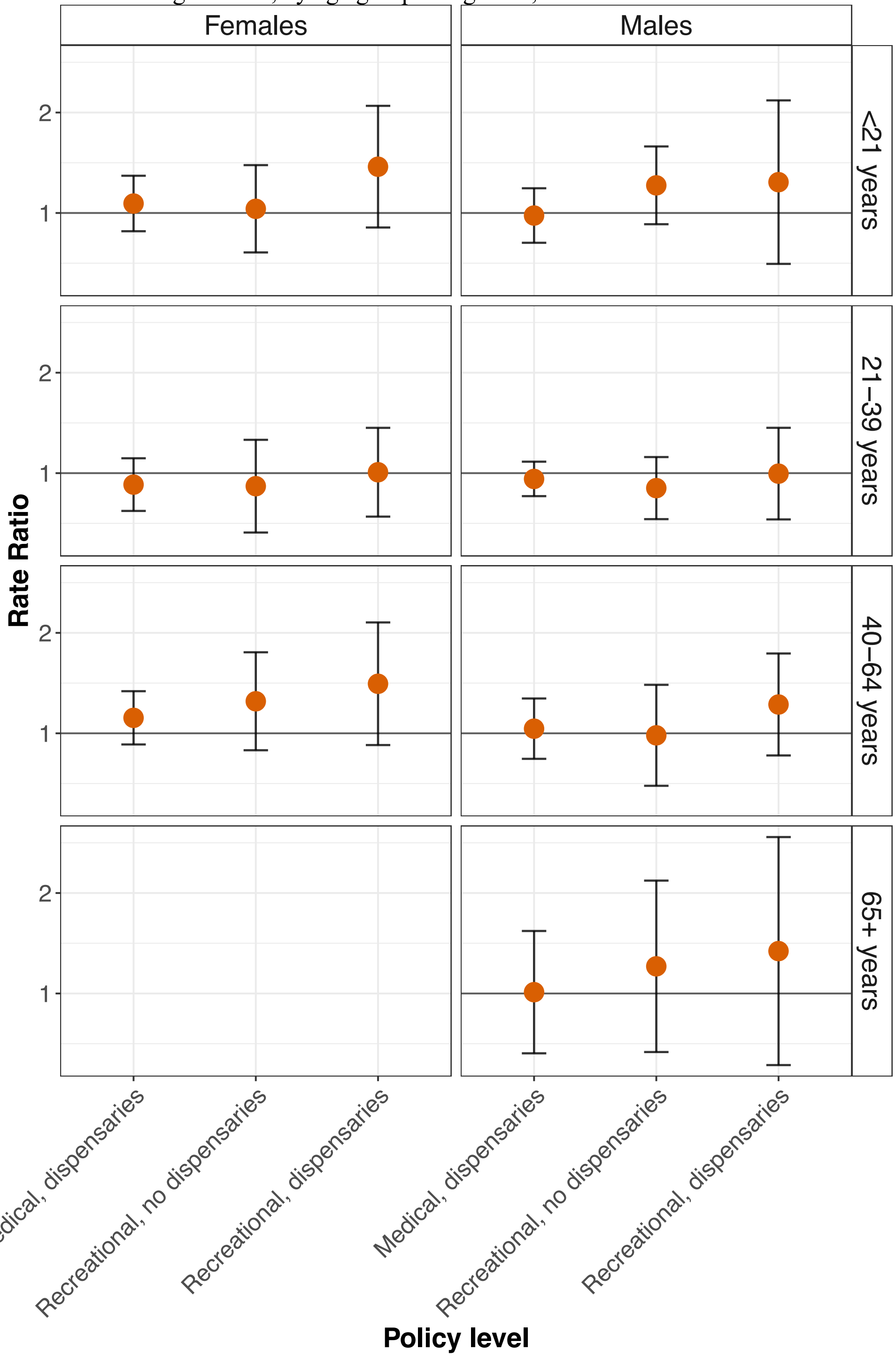

Legend: Points represent the estimated rate ratio for the association of each type of cannabis commercialization with assault injuries, relative to state-months with medical cannabis without dispensaries. The primary analysis used all state-months 2003-2017, including state-months with and without cannabis legalization laws. This analysis used only state-months in which some form of cannabis legalization was effective, with the most stringent category (medical, without dispensaries) as the reference category. Bars represent the corresponding 95% confidence intervals. Assault claims for women aged 65 and older were rare in cannabis legalization states; results for this age-gender group were unstable and are not reported.

© 2021 Matthay EC et al. *JAMA Network Open*.

eFigure 29: Adjusted associations of medical and recreational cannabis commercialization with self-harm injury rates, for states with cannabis legalization, by age group and gender, 2003-2017

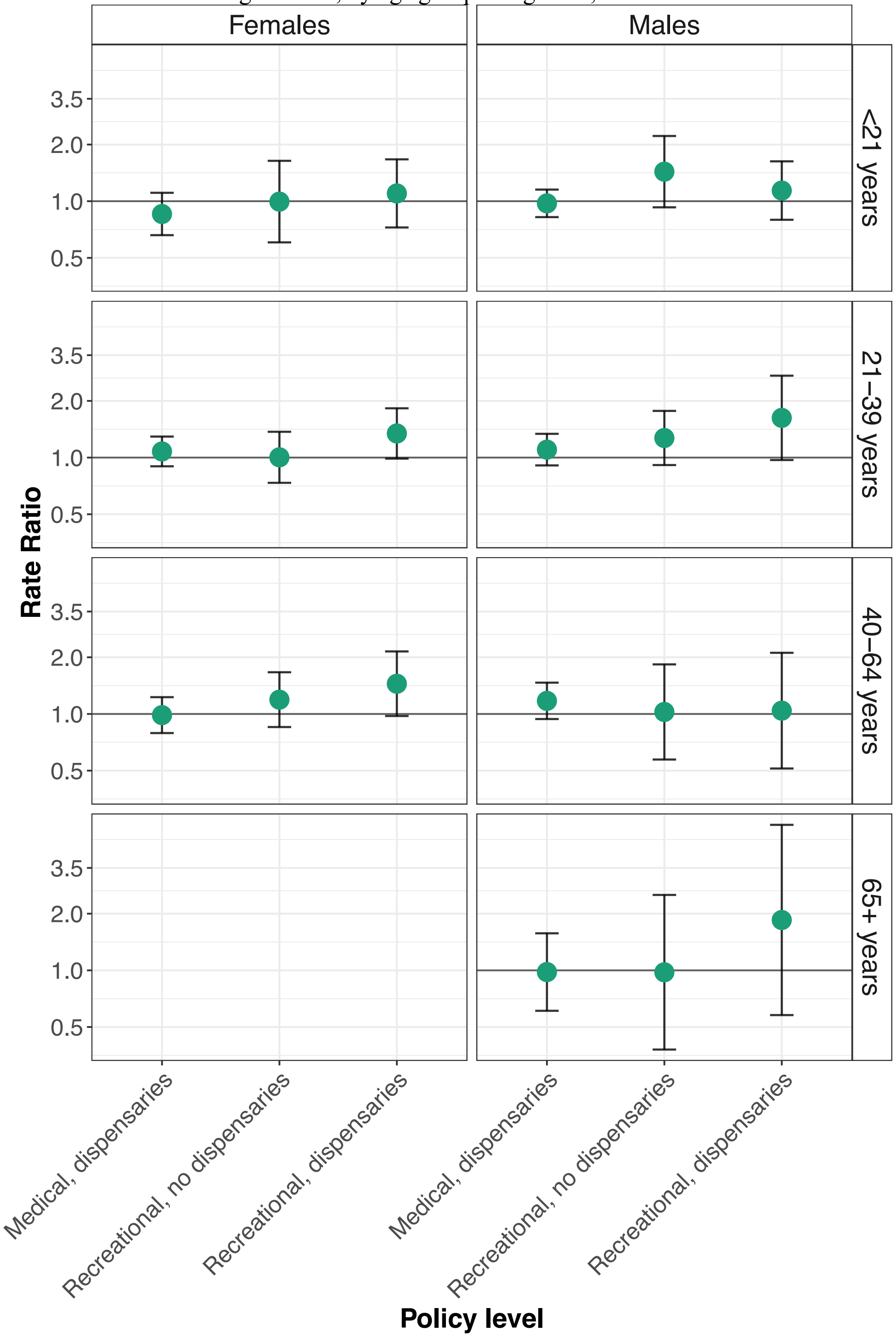

Legend: Points represent the estimated rate ratio for the association of each type of cannabis commercialization with self-harm injuries, relative to state-months with medical cannabis without dispensaries. The primary analysis used all state-months 2003-2017, including state-months with and without cannabis legalization laws. This analysis used only state-months in which some form of cannabis legalization was effective, with the most stringent category (medical, without dispensaries) as the reference category. Bars represent the corresponding 95% confidence intervals. Self-harm claims for women aged 65 and older were rare in cannabis legalization states; results for this age-gender group were unstable and are not reported.

© 2021 Matthay EC et al. *JAMA Network Open*.
